# An interactive heuristic model to test ecological and evolutionary hypotheses on incipient polyploid species

**DOI:** 10.1038/s41598-025-29286-7

**Published:** 2025-11-27

**Authors:** Juan Sebastián Schneider, Anna Verena Reutemann, Agostina Belén Sassone, Ana Isabel Honfi, Diego Hernán Hojsgaard

**Affiliations:** 1 Programa de Estudios Florísticos y Genética Vegetal, Instituto de Biología Subtropical (PEFyGV, IBS), Facultad de Ciencias Exactas Químicas y Naturales, Universidad Nacional de Misiones (UNaM), Misiones, Argentina; 2 Instituto de Botánica del Nordeste (IBONE), Facultad de Ciencias Agrarias, Universidad Nacional del Nordeste (FCA-UNNE), Corrientes, Argentina; 3 Leibniz Institute of Plant Genetics & Crop Plant Research (IPK) OT Gatersleben, Corrensstr 3, 06466 Seeland, Germany

**Keywords:** Apomixis and self-fertility, Ecological niche, Fitness, Neopolyploidy, Polyploid-demographic establishment, Unreduced gametes, Ecology, Ecology, Evolution

## Abstract

Polyploidization is associated with lineage-specific changes that promote divergence and speciation. Knowledge about the establishment of neopolyploids is fragmentary. We use an open-source multi-agent software to build a scalable easy-to-use command center for analysing complex diploid-polyploid interactions. The workspace is a multilayered environment whose eco-variables fluctuate between generations. Reproductive syndromes, recombinant/clonal inheritance and complex traits (adaptivity, niche breadth, dispersal) are used to elicit fitness values and monitor population spatial dynamics. Neopolyploidization was recurrent, polytopic and heterogeneous in time. Increasing rates of unreduced gametes accelerated the establishment of neopolyploids but removed the role of triploids. Under standard rates and heterogeneous environment, model-based evidence shows that (1) a large proportion of polyploidization events are unsuccessful, (2) parental traits and local conditions prime a loss of diploid fitness that benefited eco-geographic structured polyploid success, (3) self-fertility and apomixis improve the rate of polyploidization, and shorten the time required for demographic establishment, and (4) ecological niche shifts promote cytotype coexistence, but niche expansion favors establishment and foster cytotype displacement. The modelling framework offers opportunities for in-depth lineage-specific analyses of the spatiotemporal dynamics and evolutionary differentiation in diploid-polyploid systems.

**Supplementary Information:**

The online version contains supplementary material available at 10.1038/s41598-025-29286-7.

## Introduction

Polyploidy is a pervasive, recurrent and major force in the diversification of plant species^1,2^. Polyploidization commonly involves the merging of an unreduced gamete with a reduced one (i.e. unilateral polyploidization) or with another unreduced gamete (i.e. bilateral polyploidization) to generate an organism holding a higher ploidy than the parents^3,4^. The fate of such neopolyploid - establishment or extinction - is complex, and depends on several low-probability events involving both deterministic (mating type, gamete viability, fertility) and stochastic (dispersal, environment) variables^5–8^.

Diploids are the starting point of any new polyploid lineage, and therefore, a neopolyploid not only must overcome physiological stress and genomic shock caused by the new polyploid condition^9,10^, they also must deal with unbalanced meiosis, reduced fertility, frequency-dependent mating disadvantages and parental competition^11^. The frequency of unreduced gamete (2*n*) formation among diploid individuals determines the proportion of neopolyploids arising in any population. Rates of 2*n* gametes are variable among sexual taxa and plant families^4^. In non-hybrid diploids it does not exceed 1%, averaging about 0.6% among natural populations^12^. Model estimations of unreduced gametes frequencies match well with the published estimates when using gametic models for diploid-polyploid systems that predict ploidy frequencies in natural populations as functions of several parameters^13^. Even so, a disparity is observed between 2*n* gamete frequency estimates in natural populations and rates of neopolyploids production^4^ implying that factors other than 2*n* gametes play a role in polyploid formation.

Important traits for the demographic establishment of a neopolyploid are those related to their reproductive mode, fertility, and ecological requirements. Although polyploidization is commonly associated with reproductive changes towards self-fertility (and, to a lesser extent, apomixis or vegetative reproduction^14,15^, the type of polyploidy can differentially promote alternative reproductive strategies^16,18,20^. While self-incompatible plants require two cross-compatible individuals and produce viable seeds, self-fertilization or apomixis skip this cost and provide an advantage during establishment and dispersal of a lineage^17,19–21^. These reproductive strategies provide reproductive assurance, and their expression expectedly impacts the establishment and dispersal phases of a new lineage.

Theoretical models generally support the expectation that for successful establishment, unreduced gametes and polyploid individuals must come with a fitness advantage, either in stable conditions^22^, or during environmental changes^23–25^, and when other factors limit gene flow among cytotypes, like assortative mating, self-fertilization, clonality^23,24,26^ or restricted pollen and seed dispersal^27,28^. Currently, sensitive models with interactive components considering the main drivers for polyploid success are partially developed. Several known theoretical models have generated relevant information about the dynamics of polyploid formation (e.g.^29–32^), but in most cases used static variables, or parametrised conditions that impose reproductive isolation or exclude triploids during modelling. Deterministic models dealing with polyploidization consider slightly different attributes (e.g.^22^; Table 1). In such models, reproductive mechanisms and a triploid cytotype bridging diploid and tetraploid populations have revealed a major influence on polyploid dynamics (e.g.^13,33,34^; Table 1). Neotriploids show variable fertility with the type of endosperm development likely influencing triploid block strength^35^. Irrespective of the disbalances in chromosomal segregation and genome dosage affecting seed development^36,37^, triploids still play a crucial role in polyploidization by acting as a triploid bridge^12,34,38–40^. Recently, the field has switched towards agent-based models, whose collective interactions develops complex system-level behaviors and so, by including stochasticity, these models offer a more accurate picture of the population dynamics (e.g.^24^; Table 1). Furthermore, several current models share a computational complexity that frequently prevents the analysis of a broad spectrum of parametric values.

Environmental drivers and demographic dynamics (during emergence, establishment and persistence of polyploids) are providing a new level of resolution on the competitive interaction between cytotypes (e.g.^41^; Table 1). In nature, polyploids are often observed to have distinct and broader distribution ranges than their related diploids, frequently owing to increased trait plasticity and tolerance to environmental changes^42,43^. Niche differentiation between cytotypes is well documented among plants (e.g.^44–46^). Ecological differentiation is variable across species^47^. Although polyploids appear to be predisposed to more rapid changes in ecological requirements^48^, the pattern of climatic niche evolution (i.e. conservatism, expansion or contraction) is likely dependent on the scale of the analysis, with intraspecific lineages showing distinct post-polyploidization niche shifts^49^. Neopolyploids could have a better chance of establishing themselves in new areas without competing with their diploid progenitors. Still, polyploids are often found coexisting in sympatry with conspecific diploids^17,47,50–52^. Understanding how subtle changes in climatic requirements may provide an advantage for local establishment of polyploids can shed new insights into the geographic persistence of neopolyploids in areas where coexisting diploids are less adapted.

The model presented here aims to overcome current limitations, by integrating existing knowledge and environmental factors within a flexible framework. Furthermore, it paves the way for large-scale modeling of polyploid events in the near future by outlining the emergence, establishment, and distribution of cytotypes across environmental gradients.

The aims of this work are *i*) to test real-time effects of ecological variability on polyploidization events; *ii*) to analyse the function of alternative reproductive strategies and variable fitness on demographic establishment and range expansion of a new polyploid; and *iii*) to provide information on poorly characterised transient steps during neopolyploid establishment and diploid-polyploid coexistence. We provide a free user-friendly modelling interface adaptable to diverse biological states (age), functional traits (ploidy, reproduction, fitness, dispersal), and ecological conditions (environmental variables) to reach the proposed objectives.

**Table 1:** Summary of polyploidization models selected based on key differentiating attributes (main assumptions, parameters, and primary results).

Population and Environment	Mate system	Gamete, viability and fertility	Primary results	References
Deterministic Models; an infinite population of hermaphroditic plants				
- Non-overlapping generations. - Homogeneous environment.	- Outcrossing (gametes mate at random).	- Inviable triploids. - The fertility and viability of the tetraploid are expressed relatively to that of the diploid.	A mixed population would occur if the production of 2*n* gametes was below 17.16%. The tetraploid cytotype excludes the diploid above this limit. The frequency of 2*n* gametes necessary for exclusion of the diploid decreases as the fertility or viability of the tetraploid increases.	^22^
- Non-overlapping generations. - Homogeneous environment.	- Outcrossing (gametes mate at random). - Selfing (with inbreeding depression).	- Inviable triploids. - The fertility and viability of the tetraploid are expressed relatively to that of the diploid.	Higher fitness of tetraploids relative to diploids in random mating populations creates less restrictive conditions for tetraploid establishment. Inbreeding depression acts to counter the effects of self-fertilization. Greater selfing in tetraploids, in the absence of inbreeding depression, facilitates tetraploid establishment and reduce the minimum rate of unreduced gamete production required for tetraploids to spread to fixation.	^33^
- Non-overlapping generations. - Homogeneous environment	- Outcrossing (gametes combine freely among individuals of the same ploidy level, but inter-ploidy crosses do not occur). - Selfing.	- Viable triploids and hexaploids. - Identical fertility of all higher ploidies. - Diploid and Triploids can produce unreduced gametes.	Higher ploidies must be less fertile relative to diploids if all ploidies are to be maintained in the system. Triploid plants are never predicted to have high frequencies.	^13^
Agent-based Models; a finite population of hermaphroditic plants				
- Overlapping or non-overlapping generations. - Homogeneous environment	- Outcrossing. - Selfing (with inbreeding depression). - Asexual (Individuals can produce clonal ramets).	- Inviable triploids. - Crosses involving unreduced gametes, are viable only when the offspring is tetraploid.	A long-lived perennial life-history strategy and clonal reproduction can increase polyploid establishment (with 0–5% unreduced gametes production rate), but to ensure success polyploids need either a substantial survival advantage over diploids, high selfed-seed production, or recurrent polyploid formation. Tetraploids rarely establish when selfed-seeds are highly inviable, unless they have very high survivorship relative to diploids.	^24^
- Overlapping generations. - Variable environment.	- Selfing. - Mating choice (outcrossing gametes that unite with gametes of the same type). - The model includes density dependence.	- Viable triploids exist, though their survival probability is lower compared to diploids and tetraploids.	High self-fertilization rates and marked reproductive isolation favor system stability, long-term coexistence of multiple cytotypes, and decrease the probability of extinction for the tetraploid cytotype. Triploids influence the probability of coexistence between diploids and autotetraploids. With low environmental variance, populations tend to converge on a high abundance of either diploids or tetraploids. As environmental variance increases, both cytotypes are more likely to go extinct.	^41^
- Overlapping generations. - Variable environment.	- Outcrossing. - Selfing. - Asexual (apomixis). - Individuals in the vicinity affect plant performance.	- Viable triploids exist, though their survival probability is lower compared to diploids and tetraploids.	Neopolyploidization is recurrent, polytopic, and heterogeneous in time. Under the same environmental optimum, neither outcrossing nor low levels of selfing are sufficient to generate successful polyploidization events (SPE). If the tetraploid environmental tolerance increases relative to diploid parents, the number of successful polyploidization events rises, accompanied by a loosening of selective pressures. Polyploids tend to establish on the margins of diploid distribution when their environmental tolerance differs from that of their diploid parents. Under different ecological optima between cytotypes, tetraploids could successfully establish and coexist with diploids.	Present work

## Methods description

### Model framework

We designed a novel collective model using the open-source software NetLogo^53^ under the GPL (GNU General Public License). This model was constructed by integrating code components directly from pre-existing models^54,55^, which allowed for the incorporation of functionalities such as hybridization or the application of uniform rules across individuals. Simultaneously, we meticulously addressed critical components like gamete classes, fitness dynamics, and environmental interactions. Consequently, we built a flexible modeling environment equipped with a central command center (Supplementary Fig. 1). This interface enables precise modification of key parameters, including the frequencies of unreduced gametes (2*n*), reproductive modes (outcrossing/selfing, sexuality/apomixis), fitness and ecological properties (shift/tolerance) for each cytotype.

### The two-dimensional space

The workspace in this model was divided into patches of 0.25 m^2^ that can hold one plant at a time. The total area was arbitrarily set at 2500 m^2^, to allow for population dynamics and small-scale range expansion of cytotypes. This fine-grained landscape is introduced to better capture the interactions between reproduction, dispersal, and genotype class within a heterogeneous environment.

Environmental heterogeneity (among patch variation), interaction between individuals (neighborhood), and seed dispersal all affect population dynamics by determining the fecundity and survival of individuals^56,57^. The environment was determined by the values of three bioclimatic variables (*V*_1_-*V*_3_) and two soil variables (*V*_4_-*V*_5_). These variables were selected based on their significant contributions to intraspecific niche differentiation and plant population growth (see e.g.^45,51,58^). The chosen Bioclimatic variables were *February solar radiation* (Rad-02; *V*_1_), *Isothermality* (Bio-03; *V*_2_) and *Annual Precipitation* (Bio-12; *V*_3_), downloaded from WorldClim website at a resolution of 1 km (http://www.worldclim.com/version2^59^). The soil variables were *Available soil water capacity* at 15 cm depth (v%; *V*_4_) and *Soil organic carbon content* at 15 cm depth (g kg^−1^; *V*_5_), both downloaded from ISRIC – World Soil Information website with a resolution of 250 m (https://www.isric.org/^60^). Each raster was geolocated at Lat − 27.59, Long − 61.69, and processed in the R environment^61^. *Raster *package (v.2.0–12^62^) was used to disaggregate (through bilinear method) and crop the rasters to a grid of 100 × 100 patches that reports the value of the given raster over the given location. Due to the greater uniformity of the values for each bioclimatic variable fitted in our micro-scale workspace, soil variables have a crucial role in restricting the population distribution.

To account for intergenerational environmental variability, a temporal factor (*SV*) was introduced into the model. This represents a heterogeneous environment characterised by small variations across simulations within a parameter set. *SV* acquires a random value from a normal (Gaussian) distribution with mean = 1 and standard deviation = 10^− 3^ and it multiplies the values of *V*_1_-*V*_3_ to create mild stochastic variations (0–0.1%). The *SV* value is generated de novo for each simulation (generation) and it modifies the *V*_1_-*V*_3_ values of every spatial cell in the workspace.

### Inherent attributes of an individual in the model

#### Individual life expectancy and generation time

The life expectancy of individual plants was arbitrarily settled at 10 running cycles (with a 10% chance of dying per cycle). The generation time, understood as the interval between the occupancy of a patch and the production of offspring, is one cycle, thus targeting perennial plants with overlapping generations. Demographic growth in each cycle will contribute new individuals and generate groups of age classes (Supplementary Fig. 2). Once a seed is established in an empty and ecologically suitable patch, it will grow and reproduce in the next cycle.

#### Reproductive mode, gametes, and seed ploidy

Individuals are hermaphrodite and can take one out of three reproductive types which determine how traits are transmitted (see below): sexual outcrossers (allogamous), sexual selfers (autogamous), and apomicts (clonal seeds). Individuals can be set to produce reduced (*n* = *x* in diploids and triploids; *n* = 2*x* in triploids and tetraploids), unreduced (2*n* = 2*x* in diploids; 2*n* = 3*x* in triploids and 2*n* = 4*x* in tetraploids) or aneuploid (*n* = *x* + *y* in triploids) gametes. Viability and values for reproductive parameters are detailed below (see Model tests, reference values and parameters).

In the model, the probability of selecting a gamete-donor decreases with distance. Most seeds are formed from pollen originated from individuals located within a range of 1–5 patches, while only a very small fraction of seeds come from individuals separated by 7–10 patches (Supplementary Fig. 2). This way, the neighboring probability-driven combination of two gametes^63^ leads to the production of a seed. The ploidy (2*n*) of a seed (offspring) results from the combination of the female and male gametes originating a sexual seed, or the *x*-value of the female gamete in cases of apomictic seeds. While long distance pollen and seed dispersal can happen, most studies show that most seeds establish very close to the female progenitor plant^64^. In light of the above, the area of seed dispersal is close to the maternal parent (Supplementary Fig. 2).

### Qualities of the population in the model

#### Demographic growth and dispersal

Demographic growth occurred whenever a seed reaches an empty patch. Therefore, patch suitability is critical and seeds not reaching a free patch will be discarded.

The Total Effective Dispersal Kernel (TEDK)^65^ describes the probability of a propagule landing at a viable final location, after accounting for all dispersal vectors and all orders of dispersal. To apply the TEDK concept to seed dispersion in our model, a negative exponential function^66^ was used as a probability density function for determining the seed landing location. This function effectively captures the intuitive biological reality that the probability of a seed landing is highest close to the maternal plant and decreases with increasing distance. When two seeds arrive at an empty patch during the same cycle, the one with the highest fit to the environmental variables of the patch prevails (*E*; see below).

#### Environmental optimum, individual adaptivity and fitness

**Fig. 1:**
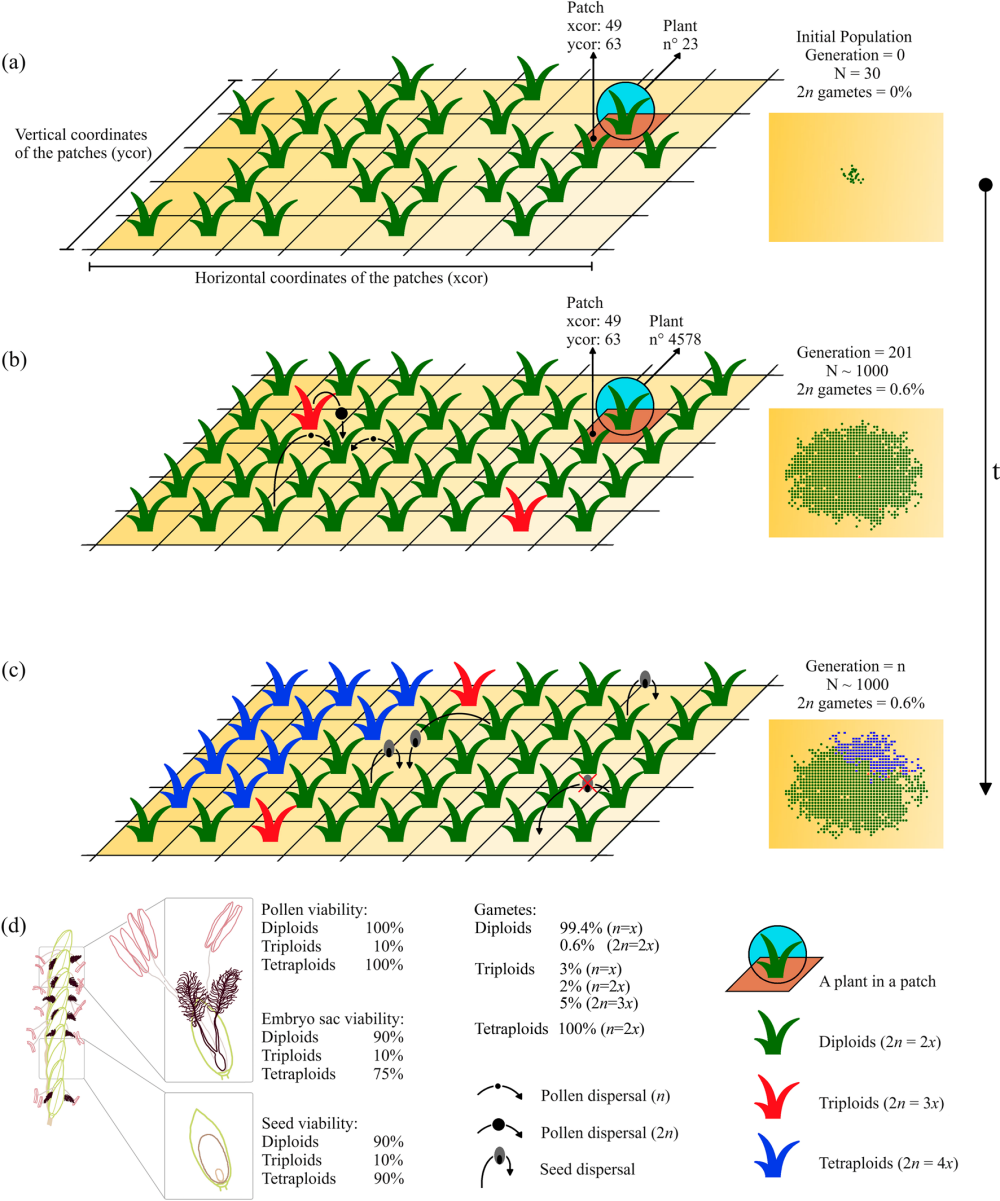
Dynamics of modeled populations (For more details see Supplementary Methods). **(a)** Initial population. Parameters for each individual plant (e.g., #23) match environmental variables of the patch, and hold *E* = 1, *Fertility* = 3 and maximum life expectancy (i.e. 10 years; Supplementary Methods). **(b)** Population at generation 201th. Individuals are cross-descendants and occupy patches according to environmental variables and the traits like adaptivity and fitness (Supplementary Fig. 4; Supplementary Methods). The values of the bioclimatic variables (*V*_1_-*V*_3_) in the patch (xcor: 49, ycor: 63) subtly change every generation, as well as age, life expectancy and *Fertility*, thus modulating the attributes of individuals and their turnover (e.g., #4578). Pollen is wind transported with a dispersal radio of 6 patches and seeds are formed base on probability density functions and proportions of reduced/unreduced gametes. **(c)** Population at *n* generations. Inherent plants attributes and emergent population attributes dominate the competitive dynamics of the population and the establishment of polyploid lineages. **(d)** Variables fixed in all the experiments after testing the model (with the exception of the percentage of unreduced gametes in diploid individuals).

At generation zero, all experiments begin with 30 individuals located in the central area of the workspace (Fig. 1). Once each plant is located, five patch-specific Individual Adaptivity values (*IA*_i_; see below) are printed, each associated to one of the five selected environmental variables (*V*_i_; Supplementary Methods). As each individual have different values corresponding to the assigned patch, the optimum of the population corresponds to a range of *V*_i_ values covering the area that includes those values in the workspace (Supplementary Fig. 3). From now on, these plants will reproduce and expand, and their offspring will inherit their attributes and create variability.

Hence, Individual Adaptivity (*IA*) as a measure of the individual´s adaptation to the environment (a genotypic attribute) is associated with the environmental background defined in the workspace (see above) by a *LimitV*_i_ (Eq. 1). *Limit* is based on the difference between *IA*_i_ and the variable *i* and it determines the “adaptation” in a patch as a shift from the optimum (Supplementary Fig. 4; Supplementary Methods).

1 $$\:E_1\:=\:\frac{1}{{2\:}^{\left|\:\right(\:IA_\text{1}\:-\:V_\text{1}\:\times\:\:SV\:)\:/\:LimitV_\text{1}\:|}}$$

Changes in the environmental component of fitness (*E*) affect the individual´s reproductive ability, thus limiting the expansion of plants. An environmental component of fitness = 1 confers an individual with maximum life expectancy (10 generations) and a maximal number of offspring, determined by age. Specifically, the maximum number of offspring for one-year-old plants is 3, for two-year-old plants is 6, and for older plants is 10 (Supplementary Fig. 3).

#### Trait inheritance

Sexual reproduction will result in offspring with varying *IA* values resulting from the average contribution of each parent (Supplementary Fig. 5). Building upon the theoretical underpinnings of quantitative genetics, our formula was expressed by adapting principles from Nicholas Barton’s infinitesimal model^67^. Our approach eliminates the potential effects of genetic drift on *IA* values within the model, as we are not simulating random fluctuations in allele frequencies. Conversely, apomixis will result in offspring with the exact same *IA* as the female progenitor. Note that our model incorporates polyploidy, but it does not account for gene-level or structural chromosomal mutations.

#### Model tests, reference values and parameters

We established a baseline using standard settings and average values from studies on natural systems (Fig. 1; Supplementary Methods). Even when a cytotype disappears, it can reappear if other cytotypes in the population generate the necessary gametes. For this reason, an emerging population of neotetraploids was considered as established when they reached 50 individuals derived from a single polyploidization event (Supplementary Table 1; Supplementary Fig. 6).

### Experiments and simulations

We focused on three types of parameter set to analyse different aspects of polyploidization events and neopolyploid establishment. Three fundamental variables were considered, (i) the frequency of unreduced gametes, (ii) the reproductive mode, and (iii) the environmental tolerance. For each case, we used baselines conditions (Fig. 1) and modified specific variables to target initials steps during the origin and demographic establishment of putative polyploid lineages. A total of 1,000 independent replicates were performed for each experiment to ensure robust estimates of lineage emergence conditions and account for stochasticity. Each replicate was set to stop when the tetraploid proportion in the population reached 1 (i.e., fixation) or when 1,000 generations had passed since diploids began producing unreduced gametes (generation 200).

#### Experiments with unreduced gametes

A range of frequencies of unreduced gametes was used to evaluate the effects of biologically realistic and unrealistic conditions. The same frequency and viability of 2*n* female and male gametes was assumed.

#### Experiments with reproductive modes

The reproductive system was treated as a constant probability parameter for all individuals in each simulation, only changing between simulations. A range of proportions of selfing complemented with outcrossing (from 0% selfing and 100% outcrossing, till 100% selfing and 0% outcrossing) and apomixis was used to evaluate the consequences of alternative breeding systems and type of inheritance (recombinant versus clonal) on the establishment and expansion of neopolyploids (see Table 2). In the apomictic pathway, the potential for apomixis in ovules was established as equivalent to the production of unreduced (2*n*) female gametes.

#### Experiments with environmental requirements

Different values for the *Limit* variable were used to either reduce, maintain, or increase the environmental tolerance of tetraploid plants relative to diploid plants, and to evaluate the effect of changing the range of ecological requirements on neopolyploid establishment. Here, all plants were considered self-sterile. In a second parameter set the optimum of tetraploids was shifted by random selection of 30 new patches distant from the initial area of diploids. *IA* values were matched to the *V*_i_ in those new patches. This way, a different environmental optimum was assigned to tetraploids compared to diploids/triploids and the inheritance of *IA* values was modified (Supplementary Fig. 5). To visualise possible effects emerging from the location of an individual in the population (central, intermediate, or peripheral), we generated a density map for all first neopolyploid individuals that resulted in SPE, and tracked the relative environmental component of the fitness of those individuals.

### Statistical analysis

Data were processed in R Studio^61^. The evaluated response variables were Successful Polyploidization Events (SPE), Unsuccessful Polyploidization Events (UPE), Bilateral polyploidization, Unilateral polyploidization and the proportion of 4*x* at the end of each experiment (detailed in Table 2). An Analysis of Variance (ANOVA) was used to compare variable means among simulations within each experiment type. Subsequently, a Tukey’s (HSD) *post-hoc* test was conducted using the *agricolae* and *stats* packages^68^. Distinct letter groupings (e.g., a versus b; Table 2) were assigned to experiments with statistically significant differences.

The model, a brief tutorial, and the necessary R code for processing the results are stored at: http://hdl.handle.net/11336/253196.

## Results

**Fig. 2:**
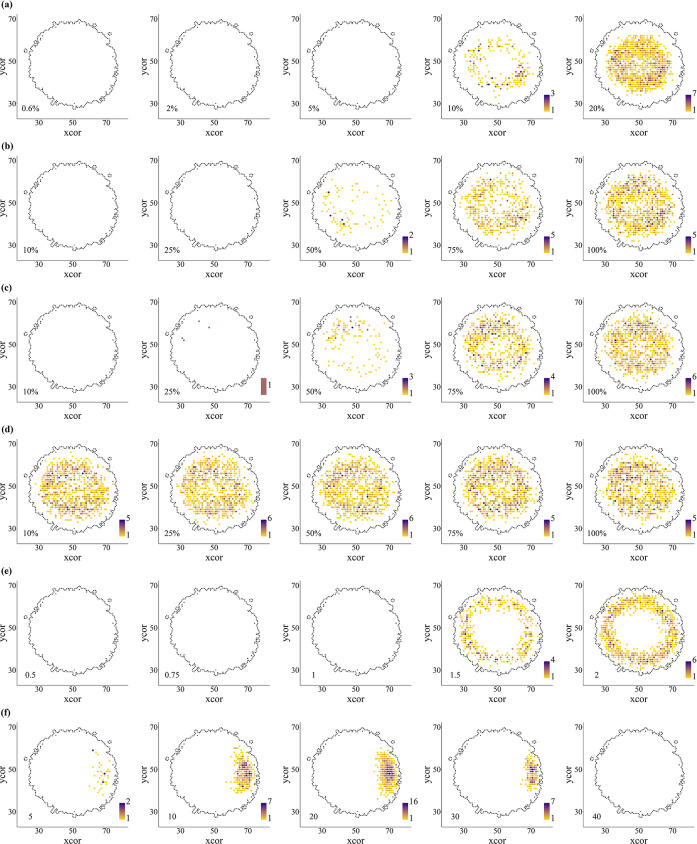
Cumulative distribution of the first successful polyploidization event (SPE) under different parameter sets. SPE locations (density-coloured dots) from 1000 replicates. Experiments using **(a)** different percentages of unreduced gametes in diploid plants; **(b)** different percentages of selfing rates in tetraploid plants; **(c)** different percentages of apomixis rates in tetraploid plants; **(d)** different percentages of apomixis and self-fertilization in tetraploid plants; **(e)** *Limit* values that reduce, maintain or increase the environmental tolerance of tetraploids relative to diploid plants; and **(f)** an environmental optimum in tetraploids different to diploid plants. xcor and ycor: horizontal and vertical coordinates of the patches. The black line delimits the maximum possible distribution of diploid individuals.

The real-time effects of the heterogeneity of ecological variables on polyploidization events were tested in a model with a command center built for this work. Most of the commands are capable of simulating natural conditions with integrated environmental variables, inherent and emergent attributes that self-modulate and determine the dynamic development of the population. Due to the randomness of pollen and seed dispersal, and the appearance of free suitable patches caused by senescence (age), each run provides evidence on the emergence time and site of polyploids following their competing abilities to the parental diploids based on the ecological background and the *IA*. Thus, the data collected about cytotype proportions found at the end of each run in all replicates provided useful information on the speed and rate of establishment of the neopolyploids, their interaction with the parental diploids and their expansion throughout the model workspace (Table 2; Fig. 2).

### Unreduced gametes experiments

When keeping all other parameters equal, increasing the frequency of unreduced gametes in the diploid cytotype increased the frequency of polyploid formation in all experiments (Table 2; Fig. 2a). Neopolyploidization was recurrent, polytopic and heterogeneous in time. Not all polyploidization events led to the establishment of a tetraploid lineage.

Under our modelling conditions, at frequencies of 0.6% unreduced gametes, diploid populations generated on average ca. 450 polyploidization events in different points of the distribution, but all were unable to reach establishment. Without other factor sustaining a relative advantage (see below), neopolyploids started to be (rarely) established only after reaching 5% of unreduced gametes production, despite a 10-fold increase in the number of polyploidization events. The gradual increase in the rate of 2*n* gametes was followed by an increase in unsuccessful polyploidization events (UPE) and a shift in the dominant polyploidization mechanism, from unilateral to bilateral polyploidization (Table 2).

The formation of partially viable and fertile triploids (Fig. 1; Supplementary Methods) skipped the ploidy barrier observed in other models. This contributed to speed up successful polyploidy event (SPE), and tetraploid fixation was possible at 2*n* = 5% but became more evident at 2*n* ≤ 10%, despite keeping UPE and generations until fixation (GuF) very high (Table 1). After this point, polyploids become more efficient and quicker to establish, reducing the number of generations required for the first SPE by 37-folds and the number of UPEs by 14-fold (Table 1). Moreover, the number of generations required until diploid displacement is shortened dramatically, from 992 generations in random runs with 10% unreduced gamete production to an average of 76 generations in runs with 20% unreduced gametes. As diploids continue to produce a basal number of unreduced gametes, the role of triploids diminishes and the chances of the first tetraploids to mate and generate new tetraploids increase. Once tetraploids started to become established, several parameters fell dramatically with the increase in 2*n* gametes’ rate (Table 1). Most relevant changes were observed in the efficiency of the mechanism (UPE; bilateral and unilateral events) and speed to fixation of polyploids (GPIP and GuF; Table 2). Thus, increasing from 10 to 20% 2*n* gametes reduced GPIP from 46 to 30 generations, suggesting a regularization of the polyploidization mechanisms (Table 2). The number of bilateral and unilateral polyploidization events showed a significative decline, with the latter having a greater influence on SPE (Supplementary Fig. 7), caused by tetraploids reaching fixation (4*x* = 1) in a much lower number of generations (as few as 76 generations; see GuF; Table 2). In addition, SPE show a significantly higher environmental component of the fitness than UPE (*p* < 0.001; Supplementary Table 2).

**Table 2:** Main parameters and statistics of polyploidization events in a multidimensional heterogeneous environment.

Parameter set		Polyploidization events						
Type (bearer ploidy)	Variable	SPE ± s.d.	GPIP	UPE ± s.d.	Bilateral ± s.d.	Unilateral ± s.d.	4*x* ± s.d.	GuF ± s.d.
Unreduced Gametes (2*x*)	2*n* = 0.6%	0.00 ± 0.00c	*na*	449.7 ± 21.5e	11.7 ± 3.4e	438.9 ± 21.1d	0.00 ± 0.00c	*na*
	2*n* = 2%	0.00 ± 0.00c	*na*	1577.7 ± 38.4c	133.2 ± 11.5d	1447.7 ± 37.0c	0.00 ± 0.00c	*na*
	2*n* = 5%	0.00 ± 0.00c	*na*	4328.2 ± 65.4b	831.7 ± 28.0b	3505.0 ± 59.4b	0.01 ± 0.00c	*na*
	2*n* = 10%	0.48 ± 1.23b	46	9388.4 ± 1006.4a	3133.6 ± 338.8a	6273.4 ± 675.4a	0.09 ± 0.22b	992 ± 59
	2*n* = 20%	9.14 ± 2.01a	30	649.3 ± 74.4d	349.1 ± 39.1c	309.4 ± 39.3e	1.00 ± 0.00a	76 ± 9
Selfing (4*x*)	10%	0.00 ± 0.00d	*na*	449.8 ± 21.3a	12.0 ± 3.5a	438.7 ± 21.1a	0.00 ± 0.00d	*na*
	25%	0.00 ± 0.00d	*na*	448.5 ± 20.9a	12.1 ± 3.5a	437.3 ± 20.4a	0.00 ± 0.00d	*na*
	50%	0.09 ± 0.30c	60	443.0 ± 37.2a	11.8 ± 3.6a	432.1 ± 36.3a	0.04 ± 0.18c	999 ± 24
	75%	0.70 ± 0.76b	61	358.8 ± 117.0b	9.4 ± 4.3b	350.6 ± 113.8b	0.45 ± 0.47b	892 ± 176
	100%	1.49 ± 0.78a	57	212.4 ± 108.2c	5.6 ± 3.6c	208.3 ± 105.4c	0.92 ± 0.25a	620 ± 220
Apomixis (4*x*)	10%	0.00 ± 0.00d	*na*	450.6 ± 20.9a	12.0 ± 3.5a	439.5 ± 20.5a	0.00 ± 0.00d	*na*
	25%	0.00 ± 0.06d	*na*	449.3 ± 21.1a	11.7 ± 3.3a	438.5 ± 21.0a	0.00 ± 0.01d	*na*
	50%	0.13 ± 0.35c	54	437.1 ± 47.8b	11.7 ± 3.7a	426.4 ± 46.6b	0.07 ± 0.24c	996 ± 38
	75%	0.77 ± 0.71b	58	339.9 ± 123.0c	8.9 ± 4.3b	332.2 ± 119.8c	0.53 ± 0.47b	864 ± 190
	100%	1.60 ± 0.84a	55	231.4 ± 111.4d	6.3 ± 3.7c	226.9 ± 108.4d	0.89 ± 0.28a	707 ± 218
Apomixis and selfing (4*x*)	10%	1.47 ± 0.76b	56	208.9 ± 110.1ab	5.6 ± 3.6ab	204.9 ± 107.3ab	0.92 ± 0.25ab	612 ± 221
	25%	1.46 ± 0.76b	54	206.1 ± 111.1bc	5.6 ± 3.8ab	202.1 ± 108.2bc	0.92 ± 0.25ab	603 ± 223
	50%	1.51 ± 0.74b	56	193.8 ± 105.0c	5.1 ± 3.3b	190.2 ± 102.5c	0.94 ± 0.21a	571 ± 218
	75%	1.48 ± 0.74b	52	200.0 ± 109.3bc	5.4 ± 3.5b	196.2 ± 106.5bc	0.93 ± 0.23ab	584 ± 224
	100%	1.72 ± 0.86a	55	221.2 ± 108.2a	6.0 ± 3.7a	217.1 ± 105.3a	0.91 ± 0.25b	678 ± 218
Environmental tolerance (4*x*)	0.5 × *Limit*_2*x*_	0.00 ± 0.00c	*na*	450.2 ± 21.3a	12.1 ± 3.6a	439.1 ± 20.9a	0.00 ± 0.00c	*na*
	0.75 × *Limit*_2*x*_	0.00 ± 0.00c	*na*	449.6 ± 20.3a	12.0 ± 3.5a	438.4 ± 20.0a	0.00 ± 0.00c	*na*
	1.0 × *Limit*_2*x*_	0.00 ± 0.00c	*na*	449.4 ± 21.7a	11.9 ± 3.5a	438.4 ± 21.7a	0.00 ± 0.00c	*na*
	1.5 × *Limit*_2*x*_	0.49 ± 0.63b	19	364.0 ± 130.8b	9.7 ± 4.6b	355.3 ± 127.2b	0.40 ± 0.47b	887 ± 206
	2.0 × *Limit*_2*x*_	1.20 ± 0.63a	18	191.6 ± 132.2c	5.1 ± 4.1c	187.7 ± 128.8c	0.92 ± 0.26a	573 ± 262
Different optimum (4*x*)	5 patches	0.04 ± 0.20d	29	447.0 ± 27.6ab	11.8 ± 3.5a	436.1 ± 26.9ab	0.02 ± 0.08d	*na*
	10 patches	0.36 ± 0.52b	23	421.0 ± 51.4c	11.0 ± 3.7b	411.1 ± 49.8c	0.16 ± 0.22b	*na*
	20 patches	0.74 ± 0.52a	16	400.6 ± 44.9d	10.8 ± 3.4b	391.4 ± 43.8d	0.34 ± 0.22a	*na*
	30 patches	0.20 ± 0.42c	14	443.4 ± 26.2b	11.7 ± 3.5a	432.8 ± 25.9b	0.09 ± 0.19c	*na*
	40 patches	0.00 ± 0.00d	*na*	449.3 ± 21.4a	11.8 ± 3.4a	438.4 ± 21.1a	0.00 ± 0.00d	*na*

**SPE**, mean number of successful polyploidization events (giving rise to a population of ~ 50 individuals) ± standard deviation (s.d.); **GPIP**, mean number of generations from the first polyploid to the first polyploid initial population (~ 50 individuals = SPE); **UPE**, mean number of unsuccessful polyploidization events ([bilateral + unilateral events] - SPE); **Bilateral**, 2*n* + 2*n* polyploidization events; **Unilateral**, 2*n* + *n* polyploidization events; **4*x***, mean proportion of 4*x* at the end of every experiment; **GuF**, mean number of generations until tetraploid fixation; ***Limit***_**2*x***_, limit of ecological tolerance of the diploids. Different letters indicate statistically significant differences (*p* < 0.01) between variables within each experiment.

### Reproductive mode experiments

When keeping all other parameters stable (2*n* gametes = 0.6% and 1.0 *Limit*_2*x*_), changing the reproductive mode had a substantial impact on SPE. Using the same environmental optimum, both outcrossing and low levels of selfing were incapable of generating SPE (Table 2). Rare SPE were detected with 50% of selfing (Table 2; Fig. 2b), indicating difficulties in the multiplication of polyploid individuals likely due to minority cytotype disadvantages (mostly non-viable mating and spatial competition). Selfing rates of 75% or higher already showed less random and steady increase in SPE, with reductions in UPE and GuF (Table 2), pointing to an intensification in the efficacy of neopolyploid establishment. Consequently, the rate of fixation of tetraploids arose ten and more than 20 times when transitioning from 50% to 75% and 100% selfing, respectively (Table 2; Fig. 2b). A similar trend was observed for outcrossing and low levels of apomixis, although the proportions of SPE and fixation of tetraploids at 50% apomixis were more than those observed for selfing (Table 2; Fig. 2c). This difference in the number of successful polyploidization events did not disappear with rates of 75% and 100% apomixis, possibly associated with positive effects of neighbors on clonal recruitment. The efficiency of successful polyploidization events was higher for both selfing and apomixis reproductive modes, especially when the environment was homogeneous for low values of the environmental component of fitness (Fig. 1). While intermediate rates of selfing or apomixis allowed cytotypes to coexist, maximum rates of selfing or apomixis in neopolyploids caused diploid extinction in a similar number of generations (Table 2; see GuF). A considerable change in the number of SPE and fixation of polyploids in the population occurred when using apomixis and selfing as mixed mating system. In such case, SPEs occurred at similar pace at all rates of apomixis and selfing, reaching a 1.7 maximum at 100% (Table 2; Fig. 2d).

**Fig. 3:**
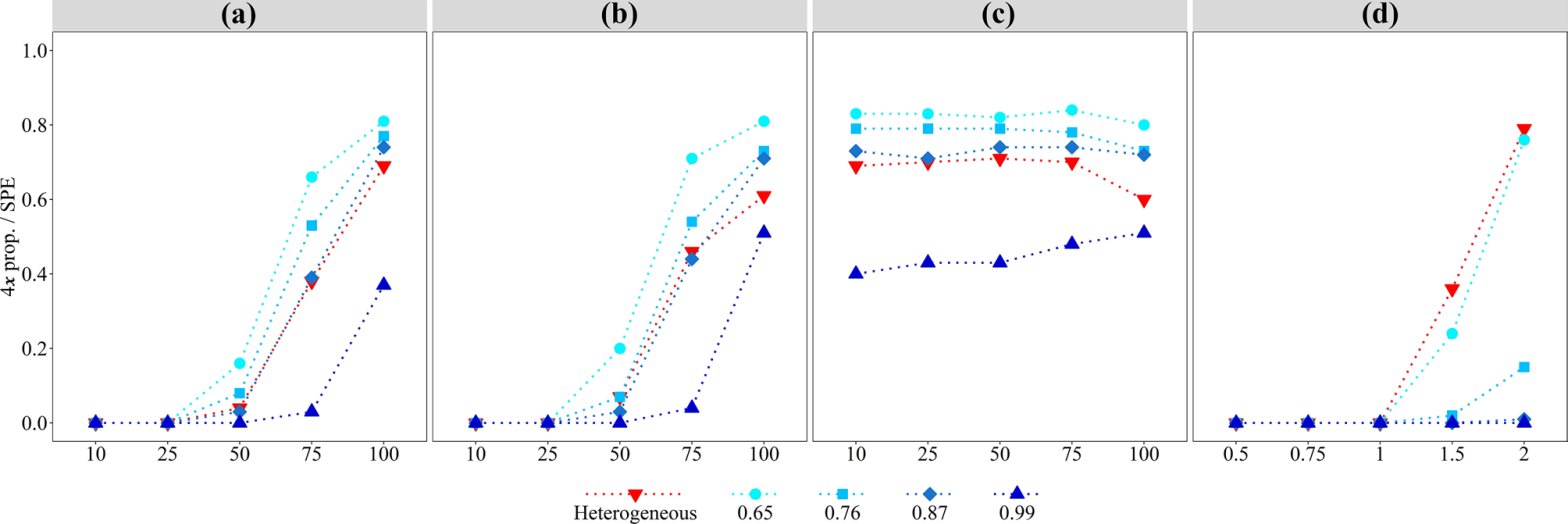
Efficiency of successful polyploidization events (4*x* prop./SPE), in heterogeneous and homogeneous environments. **(a).** Selfing (%). **(b).** Apomixis (%). **(c).** Apomixis (%) and selfing. **(d).** Environmental tolerance. Red upside-down triangles represent the heterogeneous environment. Blue circles, squares, diamonds, and triangles represent homogeneous environments established by defining values of the environmental component of fitness (*E*) at 0.65, 0.76, 0.87, and 0.99, respectively.

### Environmental sensing experiments

When keeping all other parameters equal (2*n* gametes = 0.6%; self-sterile), modifying the ecological requirements of tetraploids compared to diploids had a distinct effect. Using the same environmental optimum and increasing tetraploid environmental tolerance relative to diploid parents (*Limit*_2*x*_) was crucial for SPE. While environmental tolerance ≤ 1 showed little to no changes in all measured variables, values ≥ 1 were needed to have SPE (Table 2). When *Limit*_2*x*_ was 1.5 times greater, an average of 0.5 SPE per 1000 runs were observed (Table 2). When *Limit*_2*x*_ was 2.0 times greater the average SPE reached 1.2, shortening GPIP and GuF, and substantially increasing the rate of 4*x* fixation (Table 2; Fig. 3). Polyploids were established on the margins of diploids’ distribution (Fig. 2e), in patches where environmental values reached or exceeded the diploids’ limit of ecological tolerance. The fact that GPIP was among the lowest values, i.e. the fastest growth, among all experiments (Table 2), indicated that patch availability can promote rapid polyploid establishment. Associated to the stable frequency of 2*n* gametes, unilateral polyploidization events were dominant, with bilateral ones counting for only ca. 9% and 14% of all SPE in 1.5 and 2 *Limit*_2*x*_ runs, respectively (Supplementary Fig. 7). Surprisingly, and contrary to what was observed for all other experimental conditions, a two-fold higher tolerance provided tetraploid individuals with greater ecological flexibility and allowed them to establish with an average fitness below that observed for UPE (*p *< 0.01; Supplementary Table 2), indicating a relaxation of selective pressures.

Using different ecological optima between cytotypes showed that tetraploids became established and coexisted with diploids. A niche shift of only five patches in the grid of the workspace indicated that even a slight change in the ecological optimum of diploids and tetraploids was enough to promote SPE (Table 2; Fig. 2f). Increasing the differences of ecological optima between cytotypes reached an average of 0.74 SPE per 1000 runs around a shift between optima of 20 patches, showing low level of polyploid fixation. After this maximum, SPE decreased as the growing environmental differences segregated both cytotypes spatially (Table 2; Fig. 2f). The fitness of the first established neopolyploid is higher than the fitness of surrounding diploids (Supplementary Table 2). Although first neopolyploids can multiply and show small increases in fitness, they are below the ratio needed to avoid being outcompeted by surrounding diploids.

## Discussion

### Filling the modelling gaps

Successful establishment of a neopolyploid lineage requires the formation and concurrence of a group of intermating polyploids. Such demographic establishment represents one of the hardest phases in the evolution of a new polyploid lineage. Scientists have collected a wide range of data on the main ecological and genetic drivers that facilitate polyploid establishment, mostly from examples in established polyploids but also from experimental neopolyploids (e.g.^48,69^). Yet, much less is known about what happens in natural populations during the first generations heading to the establishment and persistence of a nascent polyploid lineage.

Traditional models evaluate such phase by generalizing static parameters or fixed environments for understanding polyploidization mechanisms (e.g.^22,70^). This often led to exclusion of relevant traits or conditions (occurrence of triploids or fluctuating environments) resulting in limited, seemingly unrealistic, outcomes. For instance, Kauai et al.^32^ used a model of spatially structured populations to reveal how neutral processes can drive to polyploid establishment. However, the model disregards reproductive and competitive interactions, and tetraploids were only formed and maintained at high rates of unreduced gamete formation (≥ 3.5%), which also enlarged displacement events.

### Unreduced gametes in sexual polyploidization: a functional component devoid for establishment

Sexual polyploidization through unreduced gametes is the leading mechanism of polyploid formation^71^. Average production of unreduced gametes across flowering plants is 0.56%^12^, but rates of 2*n* gametes varies among individuals in natural populations. Such variability is frequently represented by low/very low levels or lack of unreduced gametes in many individuals while a few shows rates over the mean. For example, in *Achillea borealis* Bong. most individuals showed occasional or no unreduced pollen production, but eight individuals had ≥ 1% unreduced pollen, with an overall average of 0.24% among 250 individuals sampled^72^. Similar patterns were detected in *Trifolium pratense* L., where 18 individuals out of 600 screened produced ≥ 1% unreduced pollen^73^, in *Dactylis glomerata* L. through interploid crosses^74^, or in *Chamerion angustifolium* (L.) Holub^75^. In rare cases, species tend to produce ≥ 1% unreduced pollen.

Environmental modulation of unreduced gamete formation^50,76^ can also provide plasticity to populations to change their ploidy and survive climatic stress. In our model-test, polyploidization events happen spontaneously in diploid populations with as less as 0.1% of unreduced gamete production. Disadvantages of minority cytotype and Allee effects due to inviable matings or pollen limitation and inverse density dependence^77^, could interfere with the demographic establishment of polyploids.

Triploids are active players during polyploidization and they enforce a considerable fitness load to the establishment of polyploids^5,34^. As the frequency of unreduced gametes rise, direct formation of new tetraploids by bilateral polyploidization becomes more likely, the probability of tetraploids concurring in close proximity to mate increases and is only restricted by seed competition for open patches. However, no data is available in plant natural population for this scheme of increased bilateral neopolyploidization frequency.

Two major implications emerge for the neopolyploidization framework. First, polyploids are unlikely to establish at the average rates of unreduced gametes observed in most plant taxa unless they acquire a competitive advantage, like higher relative fitness or by colonizing an area free of diploids. Second, it gives an important role to those rare individuals with exceptionally high frequency of unreduced gametes, for increasing polyploidization events and as catalyzers of successful establishment and persistence of polyploids locally. Therefore, the variability of unreduced gametes in natural populations poses a minimal -albeit permanent- possibility that a lineage will become polyploid. Since there are genetic components for the formation of unreduced gametes that can accumulate rapidly by recurrent selection^78,79^, but see^31^, the fact that most diploid individuals show low or no production of unreduced gametes is probably frequency-regulated. Sexual triploids are frequently sterile or have reduced fitness^5,12^, diverting resources for unreduced gamete production comes at a cost to the fertility and overall fitness of diploids. When that cost is surpassed by frequencies that allow direct formation of tetraploids, the model indicated that the displacement of diploids by tetraploids reaches a maximum below 20% production of unreduced gametes.

Similar results were reached in previous models using different conditions (e.g., lack of triploids, annuality instead of perenniality). For instance, Felber^22^ found that diploid exclusion was reached in a population when the frequency of unreduced gametes exceeded 17.2% if fertility and viability in both cytotypes was the same, or 6% if tetraploids’ fertility and viability doubled that of diploids. Our simulation using Felber’s conditions showed that tetraploid proportion increased quickly for 2*n* values higher than 10%. However, the regular formation of non-viable triploid progeny created a ploidy barrier that shrank the number of individuals in the population from ~ 1000 to ~ 150 before diploid exclusion at around 15.5% of 2*n* gametes. Also, if mating is restricted to individuals from nearby patches, the establishment of polyploids already occurred at 5% 2*n* gametes.

The above differences in diploid-polyploid dynamics between our model (maximum life expectancy of 10 generations) and Felber´s (one generation) point to the effects of traits such as perenniality or mating distance in lowering the required threshold of unreduced gamete production for polyploid success. The longer the lifespan of perennials the longer its persistence and the chance to accumulate compatible matings^24,41^. A comprehensive biogeographical analysis pointed to perenniality as an influential factor facilitating the establishment of new polyploid lineages, providing time for successful demographic establishment^80^. Thus, our data suggests that perennials perform slightly better during the initial stages of neopolyploid establishment, but a proper assessment was out of the scope of this manuscript. A valuable direction for future work would be to expand the model to incorporate vis-à-vis comparisons of life history strategies, allowing a deeper exploration of how longevity interacts with other variables and impacts on diploid-polyploid dynamics.

### Cooperative effects between forms of reproductive assurance boost the efficacy of polyploid establishment

While reproductive assurance prevails in successful polyploid lineages^30^, it is unclear whether such strategies are a consequence imposed by polyploidization or a filtering effect of selection. Both selfing and clonal reproduction (particularly apomixis) are known to mitigate mating disadvantages of neopolyploids, and the negative effects associated with the minority cytotype^11,24,33,34,41^. Under experimental conditions, such mitigation is most relevant in the early stages after polyploid formation^81,82^. Although traditional models have shown the enhancing effects of selfing on polyploid establishment (e.g.^27,70^), other studies indicate self-fertilization can act against polyploid formation if there is an initial fitness disadvantage^31^. Much less is known for clonal propagation^24,83^ or apomixis^29^.

On the one hand, this is consistent with the general view that mixed mating systems play a substantial role during demographic establishment of polyploids, but low levels of self-fertilization without fitness compensation or other advantages render a high probability of polyploid exclusion (e.g.^22,33^). On the other hand, asexuality harbors polyploid lineages in times of environmental and reproductive stress^84^. Our results support the premise that transient expression of apomixis in sexual self-fertile neopolyploids could promote the establishment and persistence of polyploid lineages^34^, and corroborates that such provisional association between forms of reproductive assurance is likely more frequent in nature than observed.

### Ecologically-driven differences modulate antagonistic interactions between cytotypes and geographically structure the neopolyploid establishment

Polyploidization emerges with several costs to the neopolyploids, particularly genome instability, improper cell divisions and fitness loss. Morpho-physiological changes associated to ploidy shifts often lead to changes in tolerance to biotic and abiotic stresses^85^. Such increased tolerance seems to be a common factor in many polyploid lineages among angiosperms^69^ likely promoting niche shifts and expansion. While ecological divergence between polyploids and diploid parentals are long known to play a role facilitating emergence of polyploid lineages^45,48^, the modelling dynamic behind polyploid establishment has not been yet fully explored.

Competition between different cytotypes may influence the outcome and duration of their coexistence. Levin and Felber models^11,22^ did not considered competition between cytotypes. Based on selfing rates, they postulated that the minority cytotype can establish and coexist if ecological requirements differ markedly between cytotypes or when migration and other genetic changes counteract their low frequency. However, at the initial transient steps of the process, cytotypes occur sharing several ecological requirements. Rodríguez^86^ used a symmetrical equilibrium between the two cytotypes and showed that they stably coexist at the same density as the niche separation increases.

We show for the first-time model specific responses to contrasting ecological properties and occurrence of spatially structured interactions between cytotypes. Our model shows that contracting the environmental tolerance of neopolyploids compared to diploids hinders SPEs (it renders all polyploidization events unsuccessful), but broadening the environmental tolerance of neopolyploids compared to diploids promotes SPEs and foster the displacement of diploids. Contrary, a shift in the environmental requirements of the neotetraploids compared to diploids promoted SPEs at rates dependent upon the degree of ecological differentiation, and tended to favor diploid-polyploid coexistence. As expected, the greater the niche differences between cytotypes, the lower the chances that one cytotype will outcompete and displace the other in its optimum zone. The number of SPEs increased as the new optimum of the tetraploids, until intercytotype competition was weaken due to spatial ecological segregation (at ~ 20 patches shift) and then, dispersal-imposed limitations to SPE. In both cases, establishment sites (and SPE) were markedly structured in space.

The spatial distribution was associated to the environmental background, the ecological requirements of each cytotype, and their emergent interaction. A previous model^41^ suggests that in relatively stable environmental conditions (low environmental variance), one ploidy level prevails over another. Here, when both cytotypes shared the same environmental optimum, under expansion of neopolyploids’ niche, SPEs occurred along with projected reductions in diploid’s environmental suitability. Thus, in diploid’s core distribution there is likely a slow dynamic turnover due to environmental stability and highly fitted, long-lived diploids. When there is a niche shift between cytotypes, SPEs occurred along projected reductions in the diploid cytotype’s environmental suitability towards the shift optimum and independently of neopolyploids’ niche expansion.

Novel insights into the spatial dynamics of polyploidization governed by ecological differences are provided together with mechanistic support to many field studies showing diverse patterns of cytotype distribution. For example, in diploid-polyploid systems (e.g.^29,45,87–89^), and polyploid-polyploid systems (see e.g.^90,91^) to studies revealing early speciation events (e.g.^32,92^). Likewise, in big populations, the spatial distribution of SPEs might provide a seed frame for biogeographic analysis of the consequences of ecological marginality gradients in the central-marginal hypothesis^93^. As a decline in ecological conditions of diploids gives opportunities for polyploid establishment and range expansions, modelling species-specific parameters and main ecological drivers can help explaining the variation in species’ demographic performance and spatial patterns of genetic variation.

### On initial fitness, recurrent origins and polyploid phylogenetics

A main strength of the model is that total fitness is not defined a priori, but arises as an emergent property of an individual’s reproductive capacity and adaptation to the environment. Thus, the fitness of diploids and polyploids varies in each generation and across space. The results indicate that while establishment requires a fitness threshold, the SPE rate and recurrent polyploidization are not strictly dependent on a specific value. Whether through fitness disadvantages or ineffective matings, neopolyploids are formed recurrently at a high homogenous pace and eliminated from diploid´s populations. The conditions associated with polyploidization override this dynamic, increasing the efficiency of demographic establishment.

The fact that SPEs consistently showed slightly higher fitness than UPEs indicates that the model setup instills an inherent selection mechanism into the ongoing biological and ecological properties. A higher fitness in neopolyploids than its surrounding diploid counterparts suggests that unfit diploid populations (perhaps due to environmental changes or expansion into suboptimal patches) have a better chance of establishing neopolyploids than fit ones. This observation also reinforces the idea of spatial structuring in SPEs. This new finding also supports the observation of increased polyploidization events in times of environmental upheaval^94,95^, but shifts the focus from the current assumption that polyploids have better chance of survival during times of environmental turmoil^69,95^ to one that bases polyploid success on diploid-polyploid interaction. Thus, since neopolyploids undergo genomic and population disadvantages like comparatively reduced fitness, polyploid success is more likely to depend on diploid fitness loss caused by temporal constraints. Our results provide a missing mechanistic link at the population level suggesting that the struggle of diploid parental species during environmental changes (to cope with fitness loss) acts as a catalyst for polyploid success. These findings add valuable information about the context of polyploid origins in genomics and phylogenetic analyses. Diploids from polyploid-containing clades are likely more vulnerable to environmental stress and prone to extinction than diploids from non-polyploid clades, which are probably more adaptive and recalcitrant to fitness loss. Integration of these dynamics can help predict populations’ responses to future climate change and extinction risks.

## Conclusions

Neopolyploidization was recurrent, polytopic and heterogeneous in time. Under heterogeneous environment the formation of neopolyploids is ubiquitous and frequently goes through a triploid bridge. Regardless of experimental setups, neopolyploids take on a population input cost of being the minority cytotype and most go extinct. Polyploidization events succeed and establish a durable population where diploids´ ecological requirements begin to be unmet, undergoes a loss of fitness, and a relative fitness advantage between diploid-polyploids becomes apparent. Thus, transiently at coexistence, the advantage of polyploids is due to the loss of fitness of diploids rather than high fitness of polyploids. These findings mechanistically support the view that diploid-polyploids are resilient systems that endure environmental turmoil and explain why polyploidization events are clustered around periods of environmental upheaval. Neopolyploidization conditions such as reproductive assurance or different ecological preferences between cytotypes increase polyploid persistence through competitive coexistence or niche displacement. Selfing and apomixis alleviate demographic establishment and promote SPEs at low rates of unreduced gametes only if coupled. Neopolyploids can coexist with diploids without ecological differentiation, although such condition relaxes intercytotype competition and favors coexistence.

## Supplementary Information

Below is the link to the electronic supplementary material.

Supplementary Material 1

## Data Availability

Our built up model and scripts are stored at: http://hdl.handle.net/11336/253196.
