# Supplementary material for: An interactive heuristic model to test ecological and evolutionary hypotheses on incipient polyploid species
Source: Sci Rep. 2025 Nov 27;16:175. doi: 10.1038/s41598-025-29286-7 (PMC12764792; doi:10.1038/s41598-025-29286-7)
Supplement: Supplementary file 1 — Supplementary Material 1 [file 41598_2025_29286_MOESM1_ESM.docx]

Article title: **An interactive heuristic model to test ecological and evolutionary hypotheses on incipient polyploid species**

Authors: Schneider J.S.; Reutemann A.V.; Sassone A.B.; Honfi A.I. & Hojsgaard D.H.

The following Supplementary information is available for this article:

**Fig. 1.** The model´s interface. This screenshot depicts most inherent individual variables for each cytotype, as well as emergent parameters and distribution of cytotypes in the population on every generation along the simulation. Note that changes in the values of the different parameters are simple and allows the user to test an uncountable number of different scenarios. The right panel shows the modeled *live* population every generation.


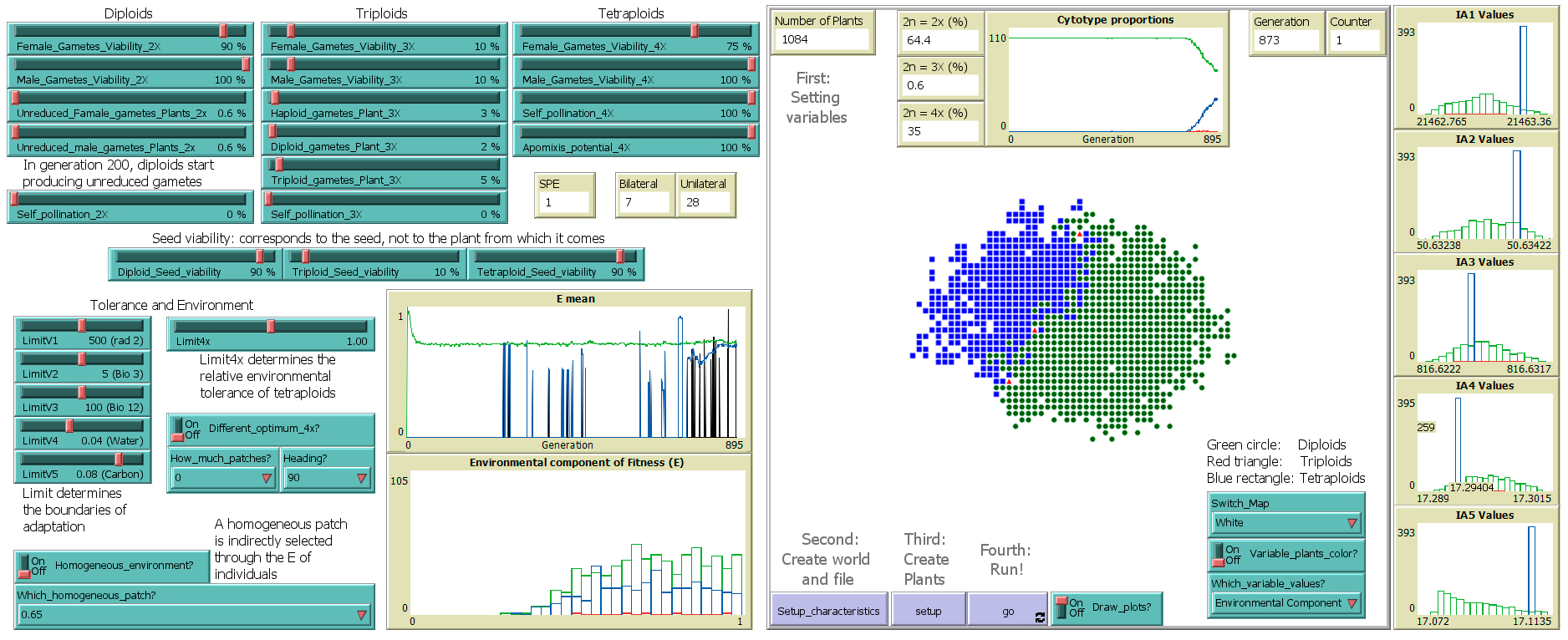


**Fig. 2.** Histogram of the different attribute frequencies in diploid individuals (at 200^th^ generation). (a). Histogram of different age classes. (b). Histogram of the frequency of pollen grains (y-axis) and dispersal patches (x-axis) depicting a right skewed unimodal trendline. (c). Histogram of the frequency of seeds (y-axis) and dispersal patches (x-axis) depicting a right skewed unimodal trendline. Pollen and seed dispersal properties were checked by tracking the origin of pollen involved in all seed-producing crosses.

**
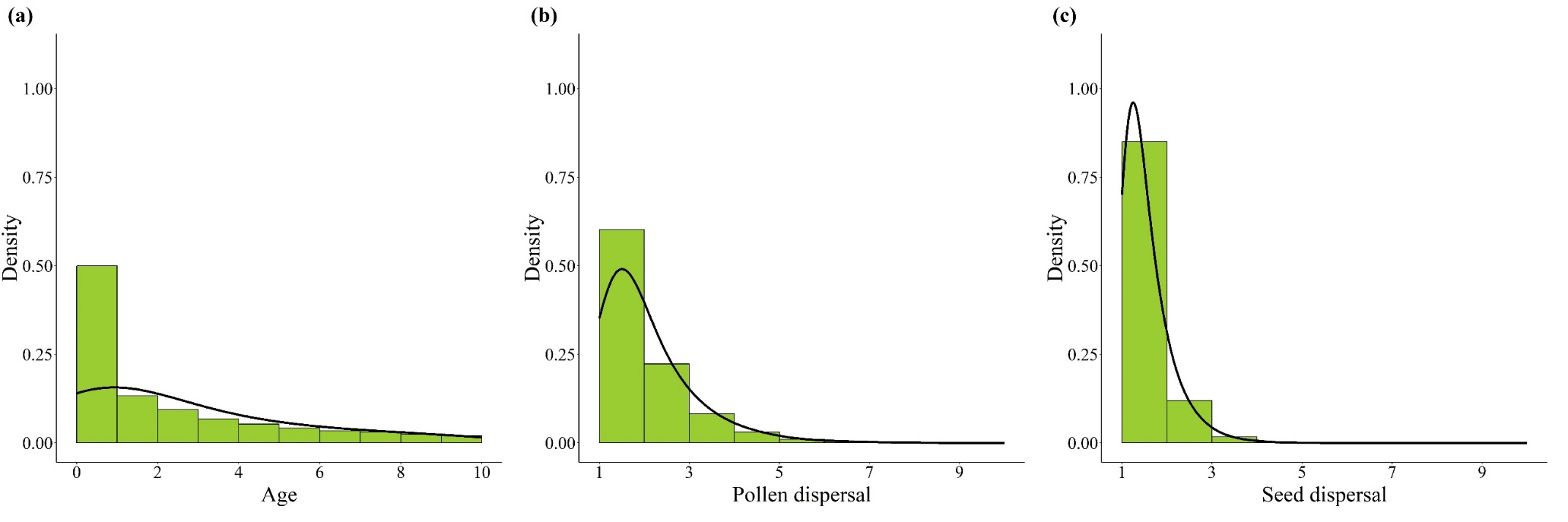
**


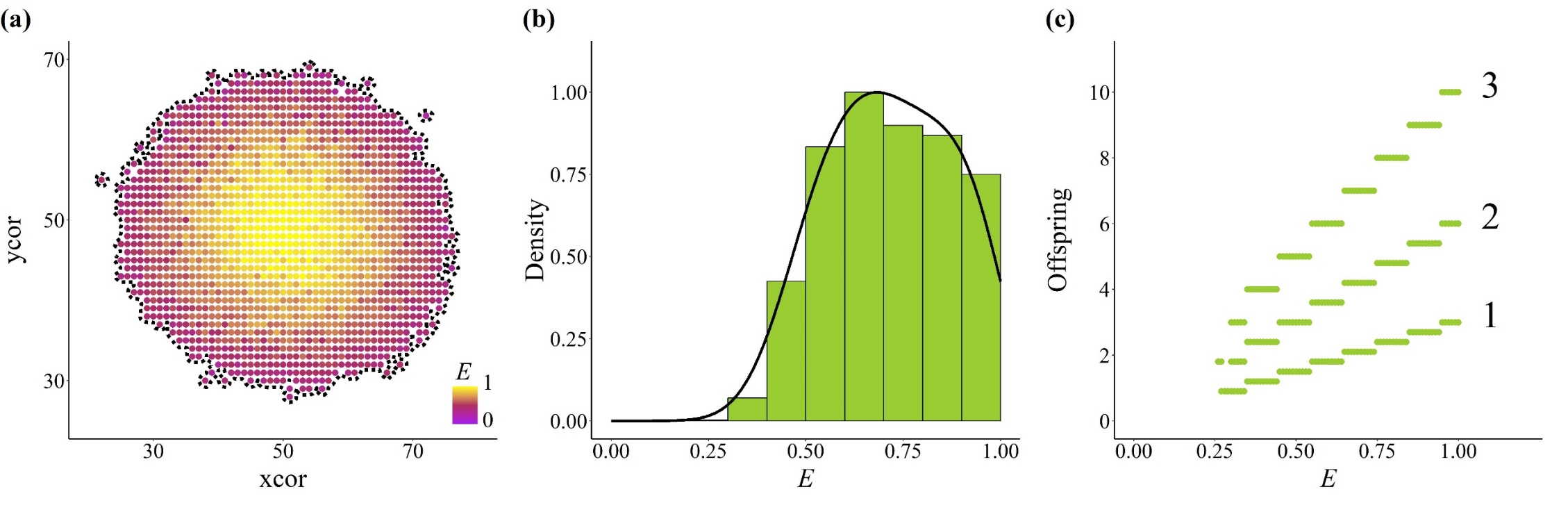
**Fig. 3.** The environmental component of the fitness (*E*) in a diploid population (at 200^th^ generation). (a). Heat map of diploid *E* values. (b). Histogram of the frequency of *E* values depicting a left skewed unimodal trendline. (c). Offspring classes ordered by *E*. Note that there is a nearly linear relationship between fitness and the number of plant offspring. The number 1 is for one-year-old plants, 2 is for two-year-old plants, and older plants are indicated with the number 3. xcor and ycor: Horizontal and vertical coordinates of the patches, respectively.

**Fig. 4.** The environmental component of the fitness (*E*_1_) of an individual along a range of *V*_1_ values. The example shows the expected values of *E*_1_ for a plant located in a patch with *V*_1_ = 1000 and *LimitV*_1_ = 500 (see details in Methods). In a year with no climatic variations (*SV* = 0), a plant with *IA*_1_ = 750 will have an *E*_1_ = 0.7. Thus, as the plant moves away from the *V*_1_ value in that patch, *E*_1_ decreases according to non-adaptive conditions.


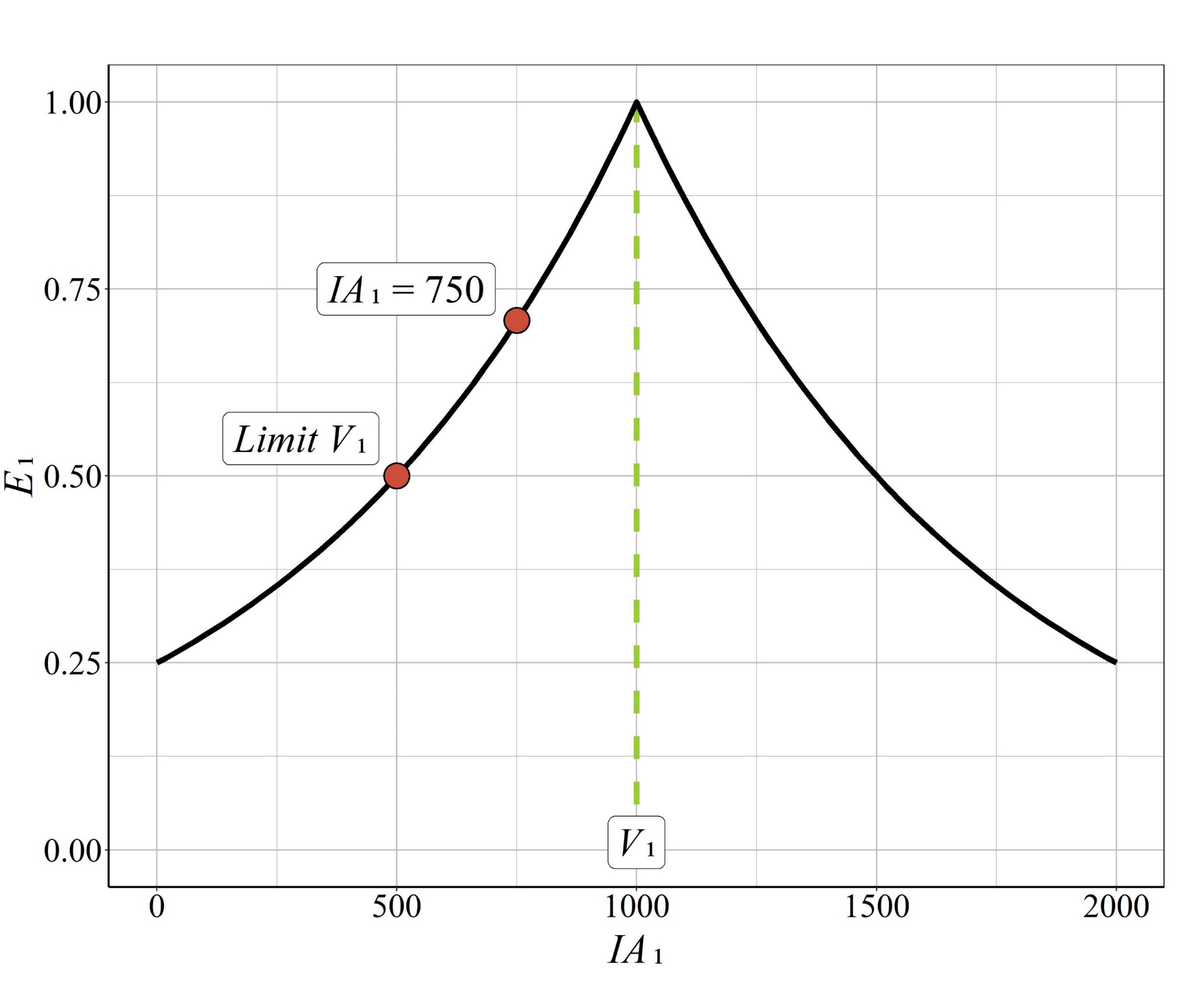


**Fig. 5.** Origin and transmission of individual adaptivity (*IA*) to offspring. Individuals from the founder population generate the initial adaptivity values of the population (black dotted line). For each individual under that distribution, a new probability distribution (blue lines) is generated using the individual´s *IA* value as the mean value (e.g., *IA*_A_ for individual A and *IA*_B_ for individual B) and defining the standard deviation (e.g., *σ*_A_ and *σ*_B_). Each time a plant reproduces, a random value within that distribution is chosen as the parental contribution to adaptivity (*PCA*; dashed red lines) following distribution probabilities. Then the *IA* of the offspring results from the average contribution of parent A and B. In the experiment of environmental optima, the optimum values vary between diploids/triploids and tetraploids, and the parental contribution to adaptivity (*PCA*) is calculated using the transformation Max*IA*_i_ *offspring* - (Max*IA*_i_ *parent*_A_ – *IA*_i_ *parent*_B_); where Max*IA*_i_ *offspring* is the maximum *IA*_i_ value for the offspring’s cytotype distribution, Max*IA*_i_ *parent*_A_ is the maximum *IA*_i_ value for the parent A cytotype distribution, and *IA*_i_ *parent*_B_ is the *IA*_i_ value of the parent B.


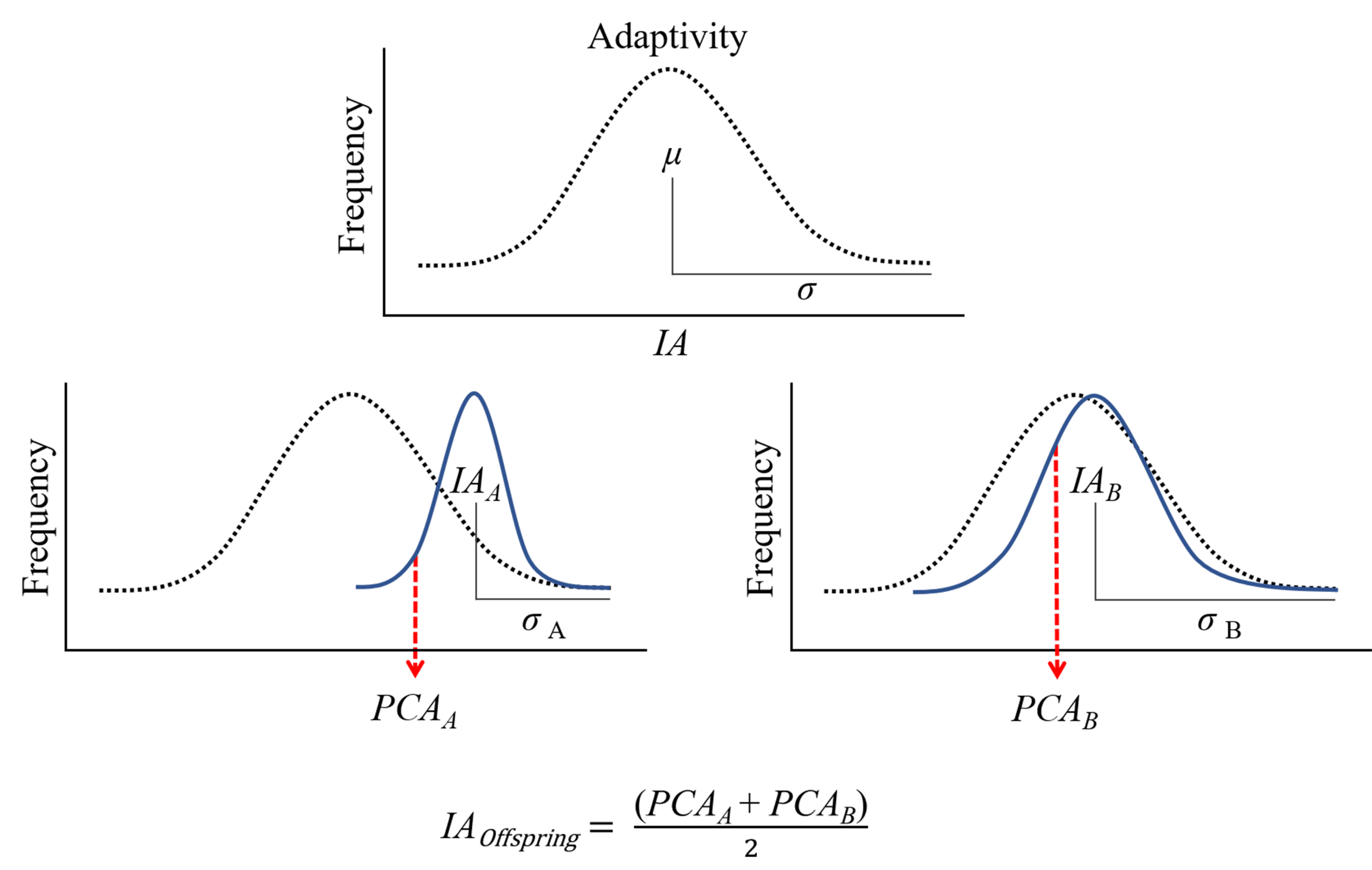


**Fig. 6.** How polyploidization events are created and how the number of polyploids associated with each polyploidization event increases. Only primary polyploids derived from diploids that produce 2*n* gametes were considered as **polyploidization events (**bilateral and unilateral**;** Supplementary Table 1). The model considered cyclic processes of neopolyploidization (increasing ploidy) and ploidy reduction. When a neopolyploid is formed (whether 3*x* or 4*x*), an ID is created. All secondary 3*x* and 4*x* individuals (Second-Generation Polyploids) will inherit the ID value from the female progenitor, unless the female progenitor lacks an ID and the male progenitor has one, in which case the ID is inherited from the male progenitor. Diploid progeny does not inherit an ID value. When 50 tetraploid plants with the same ID were present in a certain generation, a successful polyploidization event (SPE) was recorded. Any **polyploidization event** that was not part of an SPE was classified as an Unsuccessful Polyploidization Event (UPE). The arrows indicate: parental → progeny. No ID, the ID does not exist; ID Created, a new ID is created; Inherited ID, the ID value is inherited; 1°, primary polyploid; 2°, secondary polyploid.

**
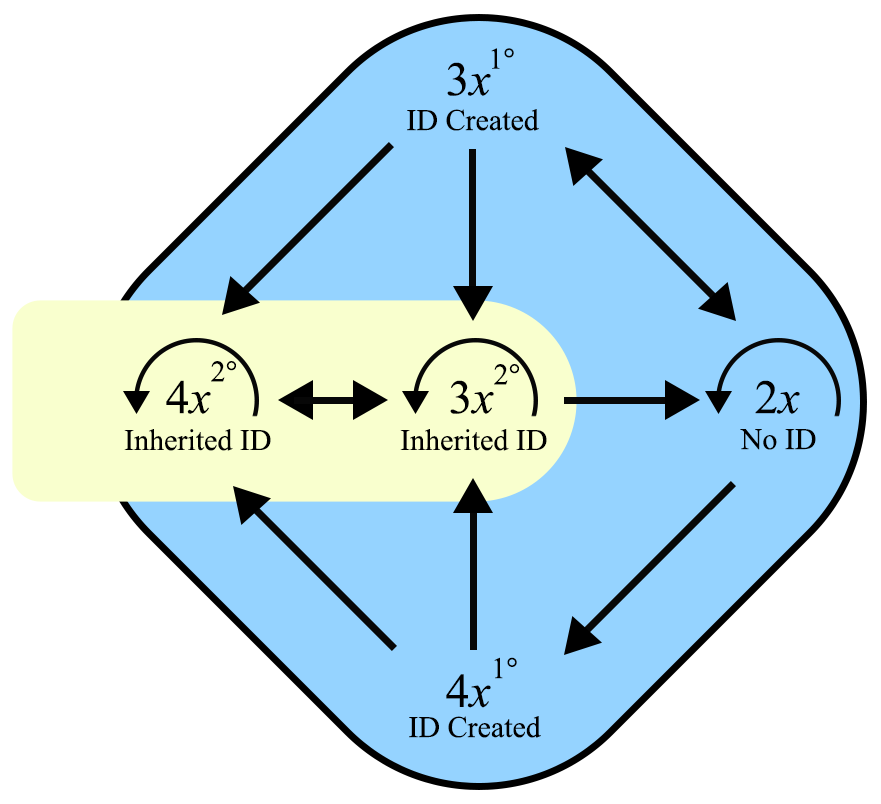
**

**Fig. 7.** Comparisons of the origin of Successful Polyploidization Events (SPE) between parameter sets. (a). Unreduced gametes (%). (b). Selfing (%). (c). Apomixis (%). (d). Apomixis (%) and selfing. (e). Environmental tolerance. (f). Different optima. Lined/blue: bilateral polyploidization events, dotted/red: unilateral polyploidization events. Total SPE are expressed in thousands, representing accumulated events after 1,000 replicates.


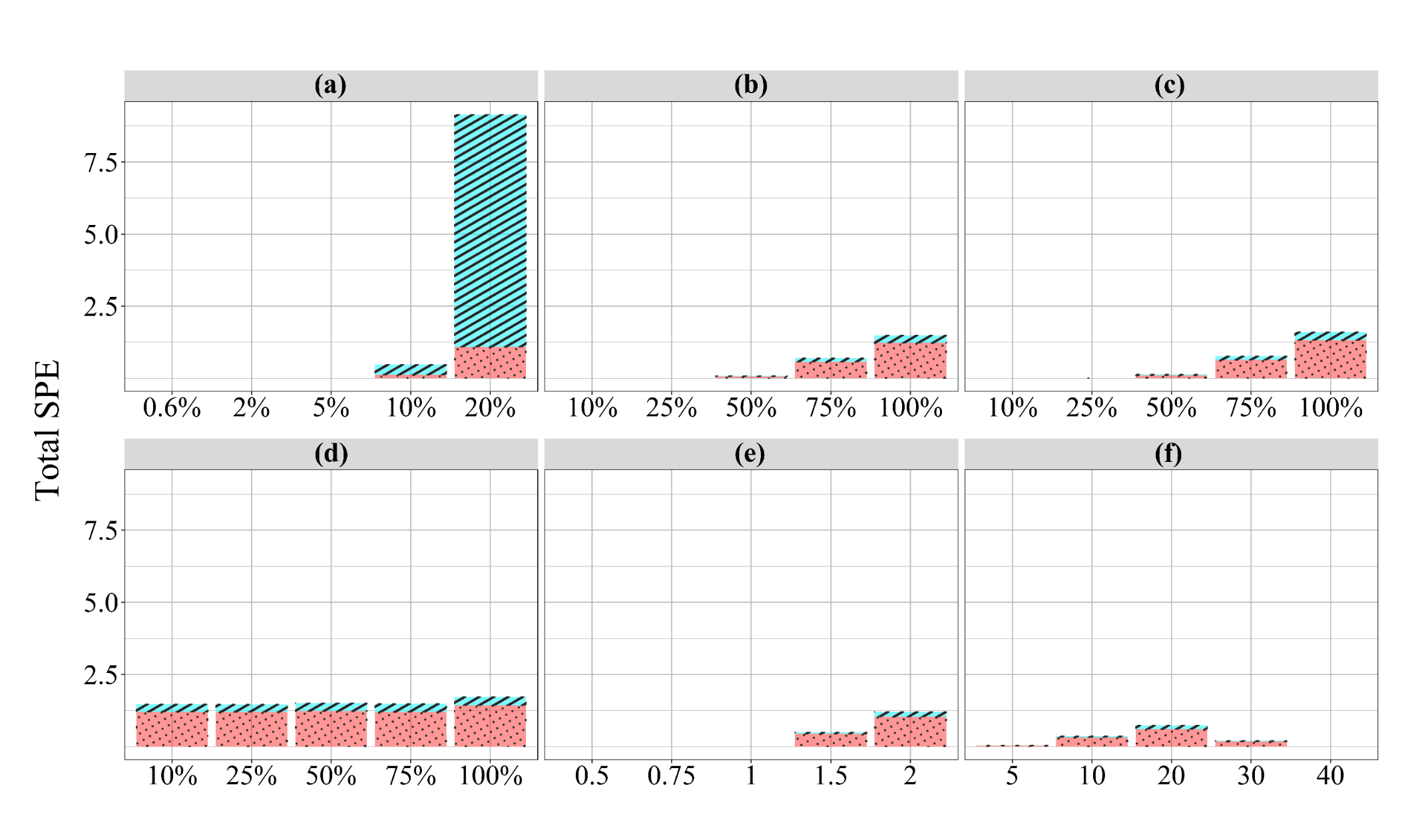


**Table 1.** Crosses and gamete contribution to neopolyploids.

|  |  |  | **Male progenitor** | | | | | |
| --- | --- | --- | --- | --- | --- | --- | --- | --- |
|  |  |  | **2*n* = 2*x*** | | **2*n* = 3*x*** | | | **2*n* = 4*x*** |
|  |  | **Gametes** | *n* = *x* | 2*n* = 2*x* | *n* = *x* | *n* = 2*x* | 2*n* = 3*x* | *n* = 2*x* |
| **Female progenitor** | **2*n* = 2*x*** | *n* = *x* | **2*x*** | **3*x*** | **2*x*** | **3*x*** | **4*x*** | **3*x*** |
|  |  | 2*n* = 2*x* | **3*x*** | **4*x*** | **3*x*** | **4*x*** | **5*x*** | **4*x*** |
|  | **2*n* = 3*x*** | *n* = *x* | **2*x*** | **3*x*** | **2*x*** | **3*x*** | **4*x*** | **3*x*** |
|  |  | *n* = 2*x* | **3*x*** | **4*x*** | **3*x*** | **4*x*** | **5*x*** | **4*x*** |
|  |  | 2*n* = 3*x* | **4*x*** | **5*x*** | **4*x*** | **5*x*** | **6*x*** | **5*x*** |
|  | **2*n* = 4*x*** | *n* = 2*x* | **3*x*** | **4*x*** | **3*x*** | **4*x*** | **5*x*** | **4*x*** |

**White**, 2*x*, 3*x*, 4*x* (**not** polyploidization events); **Blue**, 3*x* (unilateral polyploidization event [2*n* + *n*] or [*n* + 2*n*]); **Yellow**, 4*x* (bilateral polyploidization event [2*n* + 2*n*]); **Purple**, 5*x* and 6*x* (lethal).

**Table 2.** Environmental component of the fitness for SPE and UPE.

| **Parameter set** | | **All**  **FSPE±s.d.** | **First**  **FSPE±s.d.** | **First**  **FSPE Surr±s.d.** | **All**  **FUPE±s.d.** | **All**  **FUPE±s.d.(3*x*)** | **All**  **FUPE±s.d.(4*x*)** | **First**  **FUPE±s.d.(3*x*)** | **First**  **FUPE±s.d.(4*x*)** | **First**  **FUPE Surr±s.d.(3*x*)** | **First**  **FUPE Surr±s.d.(4*x*)** |
| --- | --- | --- | --- | --- | --- | --- | --- | --- | --- | --- | --- |
| **Type (Bearer ploidy)** | **Variable** |  |  |  |  |  |  |  |  |  |  |
| **Unreduced**  **Gametes (2*x*)** | 2*n* = 0.6% | *na* | *na* | *na* | 0.63 ± 0.16 | 0.63 ± 0.16 | 0.63 ± 0.16 | 0.63 ± 0.15 | 0.59 ± 0.15 | 0.66 ± 0.14 | 0.63 ± 0.12 |
|  | 2*n* = 2% | *na* | *na* | *na* | 0.63 ± 0.15 | 0.63 ± 0.15 | 0.63 ± 0.15 | 0.63 ± 0.16 | 0.66 ± 0.16 | 0.65 ± 0.14 | 0.68 ± 0.14 |
|  | 2*n* = 5% | *na* | *na* | *na* | 0.64 ± 0.15 | 0.64 ± 0.15 | 0.64 ± 0.15 | 0.64 ± 0.16 | 0.64 ± 0.16 | 0.66 ± 0.14 | 0.66 ± 0.14 |
|  | 2*n* = 10% | 0.79 ± 0.11 | 0.75 ± 0.09 | 0.74 ± 0.08 | 0.66 ± 0.15 | 0.66 ± 0.15 | 0.66 ± 0.15 | 0.64 ± 0.16 | 0.64 ± 0.16 | 0.66 ± 0.14 | 0.66 ± 0.14 |
|  | 2*n* = 20% | 0.78 ± 0.11 | 0.79 ± 0.11 | 0.78 ± 0.10 | 0.70 ± 0.15 | 0.70 ± 0.15 | 0.69 ± 0.15 | 0.65 ± 0.16 | 0.64 ± 0.16 | 0.67 ± 0.14 | 0.67 ± 0.14 |
| **Selfing (4*x*)** | 10% | *na* | *na* | *na* | 0.63 ± 0.16 | 0.63 ± 0.16 | 0.63 ± 0.16 | 0.63 ± 0.15 | 0.66 ± 0.18 | 0.65 ± 0.14 | 0.67 ± 0.16 |
|  | 25% | *na* | *na* | *na* | 0.63 ± 0.16 | 0.63 ± 0.16 | 0.63 ± 0.15 | 0.63 ± 0.16 | 0.62 ± 0.19 | 0.65 ± 0.14 | 0.64 ± 0.17 |
|  | 50% | 0.74 ± 0.12 | 0.73 ± 0.12 | 0.72 ± 0.11 | 0.63 ± 0.16 | 0.63 ± 0.16 | 0.63 ± 0.15 | 0.62 ± 0.15 | 0.68 ± 0.18 | 0.65 ± 0.14 | 0.70 ± 0.17 |
|  | 75% | 0.76 ± 0.12 | 0.75 ± 0.12 | 0.74 ± 0.11 | 0.63 ± 0.16 | 0.63 ± 0.16 | 0.63 ± 0.15 | 0.63 ± 0.16 | 0.66 ± 0.17 | 0.66 ± 0.14 | 0.69 ± 0.17 |
|  | 100% | 0.76 ± 0.13 | 0.75 ± 0.13 | 0.75 ± 0.12 | 0.63 ± 0.16 | 0.63 ± 0.16 | 0.62 ± 0.16 | 0.63 ± 0.16 | 0.60 ± 0.15 | 0.65 ± 0.14 | 0.63 ± 0.13 |
| **Apomixis (4*x*)** | 10% | *na* | *na* | *na* | 0.63 ± 0.16 | 0.63 ± 0.16 | 0.63 ± 0.16 | 0.63 ± 0.16 | 0.66 ± 0.17 | 0.65 ± 0.14 | 0.68 ± 0.15 |
|  | 25% | 0.64 ± 0.07 | 0.64 ± 0.07 | 0.64 ± 0.08 | 0.63 ± 0.16 | 0.63 ± 0.16 | 0.63 ± 0.16 | 0.63 ± 0.16 | 0.65 ± 0.14 | 0.65 ± 0.14 | 0.66 ± 0.13 |
|  | 50% | 0.76 ± 0.11 | 0.75 ± 0.11 | 0.73 ± 0.10 | 0.63 ± 0.16 | 0.63 ± 0.16 | 0.63 ± 0.16 | 0.62 ± 0.16 | 0.61 ± 0.18 | 0.64 ± 0.14 | 0.64 ± 0.16 |
|  | 75% | 0.77 ± 0.12 | 0.76 ± 0.12 | 0.75 ± 0.11 | 0.63 ± 0.16 | 0.63 ± 0.16 | 0.63 ± 0.15 | 0.64 ± 0.15 | 0.62 ± 0.20 | 0.66 ± 0.14 | 0.66 ± 0.16 |
|  | 100% | 0.77 ± 0.12 | 0.77 ± 0.12 | 0.76 ± 0.12 | 0.63 ± 0.15 | 0.63 ± 0.15 | 0.62 ± 0.15 | 0.64 ± 0.16 | 0.62 ± 0.18 | 0.66 ± 0.14 | 0.66 ± 0.16 |
| **Apomixis and**  **selfing (4*x*)** | 10% | 0.76 ± 0.13 | 0.75 ± 0.13 | 0.75 ± 0.12 | 0.63 ± 0.16 | 0.63 ± 0.16 | 0.62 ± 0.15 | 0.63 ± 0.15 | 0.60 ± 0.15 | 0.65 ± 0.13 | 0.62 ± 0.13 |
|  | 25% | 0.76 ± 0.13 | 0.75 ± 0.13 | 0.74 ± 0.12 | 0.63 ± 0.16 | 0.63 ± 0.16 | 0.62 ± 0.15 | 0.64 ± 0.16 | 0.61 ± 0.12 | 0.66 ± 0.14 | 0.63 ± 0.11 |
|  | 50% | 0.76 ± 0.13 | 0.76 ± 0.13 | 0.75 ± 0.12 | 0.63 ± 0.16 | 0.63 ± 0.16 | 0.62 ± 0.15 | 0.63 ± 0.15 | 0.60 ± 0.16 | 0.65 ± 0.14 | 0.64 ± 0.14 |
|  | 75% | 0.76 ± 0.13 | 0.76 ± 0.13 | 0.75 ± 0.12 | 0.63 ± 0.16 | 0.63 ± 0.16 | 0.62 ± 0.15 | 0.63 ± 0.16 | 0.61 ± 0.12 | 0.65 ± 0.14 | 0.63 ± 0.11 |
|  | 100% | 0.76 ± 0.13 | 0.76 ± 0.13 | 0.75 ± 0.12 | 0.63 ± 0.15 | 0.63 ± 0.15 | 0.62 ± 0.15 | 0.63 ± 0.16 | 0.63 ± 0.17 | 0.65 ± 0.14 | 0.64 ± 0.16 |
| **Environmental tolerance (4*x*)** | 0.5 × *Limit*_2_*_x_* | *na* | *na* | *na* | 0.62 ± 0.17 | 0.63 ± 0.16 | 0.22 ± 0.21 | 0.63 ± 0.15 | 0.24 ± 0.21 | 0.66 ± 0.13 | 0.67 ± 0.15 |
|  | 0.75 × *Limit*_2_*_x_* | *na* | *na* | *na* | 0.63 ± 0.16 | 0.63 ± 0.16 | 0.46 ± 0.20 | 0.62 ± 0.15 | 0.37 ± 0.20 | 0.65 ± 0.14 | 0.62 ± 0.15 |
|  | 1.0 × *Limit*_2_*_x_* | *na* | *na* | *na* | 0.63 ± 0.16 | 0.63 ± 0.16 | 0.63 ± 0.16 | 0.64 ± 0.15 | 0.63 ± 0.14 | 0.66 ± 0.14 | 0.66 ± 0.12 |
|  | 1.5 × *Limit*_2_*_x_* | 0.61 ± 0.11 | 0.60 ± 0.10 | 0.59 ± 0.08 | 0.64 ± 0.16 | 0.63 ± 0.16 | 0.81 ± 0.09 | 0.63 ± 0.16 | 0.79 ± 0.11 | 0.66 ± 0.14 | 0.64 ± 0.17 |
|  | 2.0 × *Limit*_2_*_x_* | 0.62 ± 0.13 | 0.62 ± 0.13 | 0.59 ± 0.08 | 0.64 ± 0.16 | 0.64 ± 0.16 | 0.89 ± 0.06 | 0.63 ± 0.16 | 0.90 ± 0.06 | 0.66 ± 0.14 | 0.68 ± 0.14 |
| **Different**  **optimum (4*x*)** | 5 patches | 0.65 ± 0.10 | 0.64 ± 0.10 | 0.62 ± 0.08 | 0.63 ± 0.16 | 0.63 ± 0.16 | 0.62 ± 0.18 | 0.64 ± 0.16 | 0.64 ± 0.17 | 0.66 ± 0.14 | 0.66 ± 0.12 |
|  | 10 patches | 0.63 ± 0.12 | 0.63 ± 0.12 | 0.61 ± 0.08 | 0.63 ± 0.16 | 0.63 ± 0.16 | 0.56 ± 0.24 | 0.63 ± 0.16 | 0.57 ± 0.26 | 0.66 ± 0.14 | 0.65 ± 0.15 |
|  | 20 patches | 0.63 ± 0.17 | 0.63 ± 0.17 | 0.57 ± 0.07 | 0.63 ± 0.17 | 0.64 ± 0.16 | 0.38 ± 0.27 | 0.63 ± 0.16 | 0.32 ± 0.26 | 0.65 ± 0.14 | 0.65 ± 0.13 |
|  | 30 patches | 0.57 ± 0.13 | 0.57 ± 0.13 | 0.54 ± 0.06 | 0.62 ± 0.17 | 0.63 ± 0.16 | 0.24 ± 0.23 | 0.63 ± 0.16 | 0.24 ± 0.24 | 0.65 ± 0.14 | 0.62 ± 0.11 |
|  | 40 patches | *na* | *na* | *na* | 0.62 ± 0.18 | 0.63 ± 0.16 | 0.12 ± 0.14 | 0.62 ± 0.15 | 0.12 ± 0.16 | 0.65 ± 0.14 | 0.64 ± 0.15 |

**All FSPE**, mean environmental component of the fitness values (*E*) for successful polyploidization event (SPE) ± Standard Deviation (s.d.); **First FSPE**, mean *E* values of the first SPE in each repetition; **First FSPE Surr**, mean *E* values of the SPE surrounding diploids; **All FUPE**, mean *E* values of unsuccessful polyploidization events (UPE); **All FUPE (3*x*)**, mean *E* values of 3*x* UPE; **All FUPE (4*x*)**, mean *E* values of 4*x* UPE; **First FUPE (3*x*)**, mean *E* values of the first 3*x* UPE in each repetition; **First FUPE (4*x*)**, mean *E* values of the first 4*x* UPE in each repetition; **First FSPE Surr (3*x*)**, mean *E* values of the first 3*x* UPE surrounding diploids; **First FSPE Surr (4*x*)**, mean *E* values of the first 4*x* UPE surrounding diploids. The environmental component of fitness (*E*) is calculated for each generation, and represent the *E* values for the generations in which SPE and UPE are created, respectively. These values vary slightly across generations due to *SV* (see the Methods description below).

**Methods**

**Individual Adaptivity and Fitness**

Each individual will have five *IA* values (*IA*_1_*-IA*_5_), each one associated with the environmental variables (*V*_1_*-V*_5_) of the occupied patch, and together they determine the environmental component of the fitness (*E = E*_1_×*E*_2_×*E*_3_×*E*_4_×*E*_5_). Individual Adaptivity, environmental variable and the environmental component of fitness are associated following the formula adapted from^1-2^:

$$\text{E}\text{i}\text{ }= \frac{\text{1}}{\text{2 }^{| ( \text{IA}\text{i }- \text{V}\text{i} \times\text{SV }) / \text{LimitV}\text{i} |}}$$

where *IA*_i_, Individual Adaptivity of the individual; *V*_i_, value that the environmental variable i takes in the patch; *SV*, temporal factor ; and *LimitV*_i_, the difference between *IA*_i_ and *V*_i_ that results in *E*_1_ = 0.5 (see Supplementary Fig. 4). *LimitV_i_* remains constant for each environmental variable, but slightly differs among them (following the own attributes of *V*_1_-*V*_5_). *LimitV*_i_ determines the edge value of adaptation of an individual to *V_i_* and determines the fall in *E*_i_ as it goes away from the optimum. As the difference between *IA*_i_ and *V*_i_ increases, *E*_i_ deviates from its optimal (Supplementary Fig. 4). Thus, the position of an individual in the landscape (sum of patches) will affect the seed set, as well as, its life expectancy (Supplementary Fig. 3).

**Model tests and the use of reference values and parameters**

The baseline experiments were run in a processor Intel Core i7-1165G7 2.80GHz 11th Gen, with 4 cores, a 16 GB RAM and an SSD NVMe Intel SSDPEKNW512G8H (512 GB), using an OS Windows 11 Home. The required time for each parameter set (1000 runs of 1000 generations each) was 3-4 h depending upon the experiment, and parallelization allowed several experiments to run simultaneously.

The dynamics of polyploidization events over 10 generations were checked in a simulated environment free from adaptability restrictions. Based on current literature data, a percentage of 0.6% of unreduced gamete (2*n* = 2*x*) formation^3-4^ was considered. Populations originating from a selfer individual showed a noticeable accumulation of triploids, reaching around 14 triploids per 10,000 accumulated diploid individuals. On the other hand, only around one tetraploid was observed per 10,000 accumulated diploids. Populations originating from two outcrossers individuals performed similarly in terms of triploid accumulation, but tetraploid accumulation was comparatively higher, reaching approximately 2 tetraploids per 10,000 accumulated diploids. In both populations, the first polyploid arose randomly in different generations.

Since our goal was not to explore the dynamics of higher ploidies, all gametes from tetraploids were considered as reduced (*n* = 2*x*). Male and female gametophytes were considered full compatible irrespective of the ploidy, but with different viability between cytotypes. Gametes viability is often diverse among plants and high pollen/ovule ratios are the norm^5-6^. In running tests and experiments, male and female gamete viability of diploids was set as 100% and 90%, respectively. Male viability of tetraploids was set the same as for diploids but female viability was set at 75% (Fig. 1) as is expected to be lower^7^.

Triploid plants are crucial for polyploid establishment but show internal (e.g., unbalanced chromosome segregation) and external (e.g., minority cytotype disadvantages) constraints^8^. Following different studies on natural and experimental triploids^3-4,9-10^, we set triploid´s gametes viability to 10% euploid gametes, i.e., 3% haploids (*n* = *x*), 2% diploids (*n* = 2*x*), and 5% triploids (2*n* = 3*x*) (Fig. 1). The remaining gametes were set as unbalanced (aneuploid) and non-viable. By doing so, we try to have a most simple and accurate approach to what is expected in nature, but we discarded the likelihood of the natural interference that an aneuploid gamete may create during the mating process, and the fact that, many aneuploids can at least survive some generations in nature.

Regarding the mating types, variations in the rates of outcrossing or selfing due to internal (e.g., pleiotropy, stress) or external (e.g., menthor effects, seasonal changes) factors are often not dramatic (see e.g. ^11-13^). Here, we settle obligate rates for outcrossing or selfing as baseline, but modified these values along the distinct experimental tests to evaluate trends and establish confidence intervals during formation, demographic establishment and expansion of neopolyploid individuals.

Following different studies on seed germination and seedling establishment^11,14^, seed viability was set according to its respective ploidy, which was 90% for both diploid and tetraploid seeds, and 10% for triploid ones (Fig. 1).

**Population dynamics in the model**

Simulations started (generation 0) with a group of sexual, self-sterile diploid individuals (N = 30). These 30 plants were positioned at the center of the workspace using random values (xcor, ycor) obtained from a normal distribution with *µ* = central patch value and *σ* = 2 ([Fig. 1](#Fig26)a). The group was allowed to outcross randomly through reduced gametes (*n* = *x*) until generation 200, when they began producing unreduced gametes (2*n* = 2*x*). Since we expect variation in *IA*, the goal of delaying the formation of unreduced gametes was to increase the number of diploid individuals in the population and create heterogeneity in fitness and age classes.

Outcrossing happens by the combination of two gametes from nearby patches to form a seed. Parameters for each individual plant match environmental variables of the patch (*IA*_1_-*IA*_5_ = *V*_1_-*V*_5_). These values will represent the optimal environment for the population (i.e. the range of *V*_i_ values; Supplementary Fig. 5). In the example presented in [Fig. 1](#Fig26)a, plant #23 is one of the 30 initial individuals (age = 0), with an environmental component of the fitness *E* = 1, a maximum life expectancy (i.e. 10 years), and the maximum number of offspring for its age (3; provided the seed lands in an empty and appropriate patch). The individual adaptivity (*IA*_i_) of plant #23 coincides with the value of the variables (*V*_i_) of the occupied patch (e.g., *IA*_1_ = *V*_1_; Solar radiation in February in patch [xcor: 49, ycor: 63] = 21400 kJ m^-2^ day^-1^).

After several cycles (i.e., generations), the population will consist of individuals descended from crosses (Fig. 1b). Individuals at the 201th generation are cross-descendants and occupy patches according to environmental variables and their Individual Adaptivity (Supplementary Fig. 3). In the example presented in Fig. 1b, the Individual Adaptivity values for plant #4578 resulted from the contributions of its parents for each trait (*IA*_1_-*IA*_5_; Supplementary Fig. 5). Since the environment is heterogeneous, the environmental component of the fitness of an individual will depend on the patch it occupies and will generally be less than 1. This means that the life expectancy and the number of offspring expected for that descendant in the next generation may be lower than its potential. Thus, the distribution of plants is conditioned by the individuals' Adaptivity values.

In each cycle, the value of the environmental variables (*V*_1_-*V*_3_) is modified by the temporal factor (*SV*) in all patches (e.g., in this generation *SV* = 0.001; *V*_1_ in patch [xcor: 49, ycor: 63] = 21400 kJ m^-2^ day^-1^ × 1.001). In this generation, some triploid plants may be found. In the example, a diploid plant receives reduced pollen (*n* = *x*) from two diploid plants and unreduced pollen (2*n* = 3*x*) from a triploid plant (Fig. 1b).

As generations pass, the proportion of tetraploid plants originating from crosses with viable 2*n* gametes increases within the population (Fig. 1c). Patch occupation can vary in each cycle, and seeds from surrounding plants compete with each other (seed dispersal radius ~ 1 patch). Often, a seed reaches an empty and appropriate patch, leading to the growth of a new plant. Simulation runs until 1,000 generations have passed since diploid plants began producing unreduced gametes (generation 200), or until the proportion of tetraploids in the population reaches 1 (i.e., tetraploids become fixed).

**REFERENCES**

1. Gavrilets, S. & Vose, A. Dynamic patterns of adaptive radiation. *Proceedings of the National Academy of Sciences*, **102**(50): 18040-18045 (2005).

2. Gavrilets, S. & Vose, A. Case studies and mathematical models of ecological speciation. 2. Palms on an oceanic island. *Molecular Ecology*, **16**(14): 2910-2921 (2007).

3. Ramsey, J. & Schemske, D. W. Pathways, mechanisms, and rates of polyploid formation in flowering plants. *Annual Review of Ecology and Systematics*, **29**(1): 467-501 (1998).

4. De Storme, N. & Geelen, D. Sexual polyploidization in plants–cytological mechanisms and molecular regulation. *New Phytologist*, **198**(3): 670-684 (2013).

5. Cruden, R. W. Pollen-ovule ratios: a conservative indicator of breeding systems in flowering plants. *Evolution*, **31**: 32-46 (1977).

6. Preston, R. E. Pollen‐ovule ratios in the Cruciferae. *American Journal of Botany*, **73**(12): 1732-1740 (1986).

7. Castro, M., Celeste Dias, M., Loureiro, J., Husband, B. C. & Castro, S. Competitive ability, neopolyploid establishment and current distribution of a diploid–tetraploid plant complex. *Oikos*, **2024**: e09949. (2024).

8. Hojsgaard, D. H. Transient activation of apomixis in sexual neotriploids may retain genomically altered states and enhance polyploid establishment. *Frontiers in Plant Science*, **9:** 230 (2018).

9. Kuspira, J., Bhambhani, R. N., Sadasivaiah, R. S. & Hayden, D. Genetic and cytogenetic analyses of the A genome of *Triticum monococcum*. III. Cytology, breeding behavior, fertility, and morphology of autotriploids. *Canadian Journal of Genetics and Cytology*, **28**(5): 867-887 (1986).

10. Hojsgaard, D. H., *et al*. Emergence of apospory and bypass of meiosis via apomixis after sexual hybridization and polyploidization. *New Phytologist*, **204**(4): 1000-1012 (2014).

11. Hojsgaard, D. H., Martínez, E. J. & Quarin, C. L. Competition between meiotic and apomictic pathways during ovule and seed development results in clonality. *New Phytologist*, **197**(1): 336-347 (2013).

12. Hojsgaard, D. H. & Hörandl, E. A little bit of sex matters for genome evolution in asexual plants. *Frontiers in Plant Science*, **6**: 82 (2015).

13. Reutemann, A. V., *et al*. Uniparentality: Advantages for range expansion in diploid and diploid-autopolyploid species. *Botanical Journal of the Linnean Society*, **200**(4): 563-585 (2022).

14. Karunarathne, P., Feduzka, C. & Hojsgaard, D. H. Ecological setup, ploidy diversity, and reproductive biology of *Paspalum modestum*, a promising wetland forage grass from South America. *Genetics and Molecular Biology*, **43**(1): e20190101 (2020).
